# Connexin 43 Astrocytopathy Linked to Rapidly Progressive Multiple Sclerosis and Neuromyelitis Optica

**DOI:** 10.1371/journal.pone.0072919

**Published:** 2013-08-22

**Authors:** Katsuhisa Masaki, Satoshi O. Suzuki, Takuya Matsushita, Takeshi Matsuoka, Shihoko Imamura, Ryo Yamasaki, Makiko Suzuki, Toshihiko Suenaga, Toru Iwaki, Jun-Ichi Kira

**Affiliations:** 1 Department of Neurology, Neurological Institute, Graduate School of Medical Sciences, Kyushu University, Fukuoka, Japan; 2 Department of Neuropathology, Neurological Institute, Graduate School of Medical Sciences, Kyushu University, Fukuoka, Japan; 3 Department of Neurological Therapeutics, Neurological Institute, Graduate School of Medical Sciences, Kyushu University, Fukuoka, Japan; 4 Department of Neurology, Hamamatsu University School of Medicine, Hamamatsu, Japan; 5 Department of Neurology, Tenri Hospital, Tenri, Japan; Research Inst. of Environmental Med., Nagoya Univ., Japan

## Abstract

**Background:**

Multiple sclerosis (MS) and neuromyelitis optica (NMO) occasionally have an extremely aggressive and debilitating disease course; however, its molecular basis is unknown. This study aimed to determine a relationship between connexin (Cx) pathology and disease aggressiveness in Asian patients with MS and NMO.

**Methods/Principal Findings:**

Samples included 11 autopsied cases with NMO and NMO spectrum disorder (NMOSD), six with MS, and 20 with other neurological diseases (OND). Methods of analysis included immunohistochemical expression of astrocytic Cx43/Cx30, oligodendrocytic Cx47/Cx32 relative to AQP4 and other astrocytic and oligodendrocytic proteins, extent of demyelination, the vasculocentric deposition of complement and immunoglobulin, and lesion staging by CD68 staining for macrophages. Lesions were classified as actively demyelinating (n=59), chronic active (n=58) and chronic inactive (n=23). Sera from 120 subjects including 30 MS, 30 NMO, 40 OND and 20 healthy controls were examined for anti-Cx43 antibody by cell-based assay. Six NMO/NMOSD and three MS cases showed preferential loss of astrocytic Cx43 beyond the demyelinated areas in actively demyelinating and chronic active lesions, where heterotypic Cx43/Cx47 astrocyte oligodendrocyte gap junctions were extensively lost. Cx43 loss was significantly associated with a rapidly progressive disease course as six of nine cases with Cx43 loss, but none of eight cases without Cx43 loss regardless of disease phenotype, died within two years after disease onset (66.7% vs. 0%, *P*=0.0090). Overall, five of nine cases with Cx43 loss and none of eight cases without Cx43 loss had distal oligodendrogliopathy characterized by selective myelin associated glycoprotein loss (55.6% vs. 0.0%, *P*=0.0296). Loss of oligodendrocytic Cx32 and Cx47 expression was observed in most active and chronic lesions from all MS and NMO/NMOSD cases. Cx43-specific antibodies were absent in NMO/NMOSD and MS patients.

**Conclusions:**

These findings suggest that autoantibody-independent astrocytic Cx43 loss may relate to disease aggressiveness and distal oligodendrogliopathy in both MS and NMO.

## Introduction

Multiple sclerosis (MS) and neuromyelitis optica (NMO) are inflammatory demyelinating diseases of the central nervous system (CNS). The pathological hallmark of MS is sharply demarcated demyelinating plaques with the relative preservation of axons, suggesting autoimmune responses target CNS myelin. In contrast, NMO shows selective and severe attacks of both axons and myelin of the optic nerves and spinal cord, resulting in necrotic cavitation. Although the nosological position of NMO has long been a matter of debate, the discovery of specific immunoglobulins (IgGs) against NMO, designated NMO-IgG [1], indicates NMO is distinct from MS with a fundamentally different aetiology. NMO-IgG recognizes aquaporin-4 (AQP4) [2], which is strongly expressed on astrocyte foot processes at the blood-brain barrier (BBB) [3]. Autopsied NMO cases show a loss of AQP4 immunostaining in inflammatory lesions, whereas AQP4 expression increases in demyelinating plaques in MS [4,5]. NMO-IgG/anti-AQP4 antibodies are cytotoxic to astrocytes *in vitro* and *in vivo* in the presence of complement [6–13]. Thus, the vasculocentric deposition of complement and immunoglobulins in NMO lesions [14] may represent a humoral immune attack against AQP4 on astrocytes leading to AQP4 loss. This was initially postulated to occur specifically in NMO in humans [4,5]. However, we and others recently demonstrated the extensive loss of AQP4 in active lesions of Baló’s disease [15], and diffuse [16] or patchy loss of AQP4 [17,18] in actively demyelinating MS lesions. These findings suggest that astrocytic damage as assessed by AQP4 loss may be a common denominator in heterogeneous human demyelinating conditions, including NMO, Baló’s disease and MS, especially when huge demyelinating lesions are formed [19]. However, AQP4-deficient mice do not develop demyelination [20], but rather show mitigation of experimental autoimmune encephalomyelitis (EAE) [21]. Thus, it remains to be elucidated how astrocytopathy can induce widespread demyelination.

Recently, we reported the extensive loss of connexins (Cxs) 43, 32 and 47 in demyelinated and myelin-preserved layers of acute lesions from patients with Baló’s concentric sclerosis, an extremely rare demyelinating disease [22]. Cxs form homotypic or heterotypic gap junctions (GJs) between astrocytes, or between astrocytes and oligodendrocytes. GJs appose two cells and form channels for direct intercellular communication through which intracellular second messengers, such as calcium ions and other small molecules, are exchanged. Experimentally, astrocytic Cx43 and Cx30, oligodendrocytic Cx32 and Cx47, and astrocytic Cx43 and oligodendrocytic Cx32 double-knockout mice show diffuse demyelination [23–25], suggesting critical roles of astrocytic and oligodendrocytic Cxs in maintaining CNS myelin. Astrocytic and oligodendrocytic Cxs have not been extensively studied in acute lesions of either NMO or MS while a recent report described the loss of Cx32 and Cx47 in chronic MS lesions [26]. Therefore, we aimed to clarify Cx alterations in acute and chronic demyelinating lesions from MS and NMO patients, by systematic investigation of the expression of Cxs relative to those of other astrocytic proteins, the extent of demyelination, vasculocentric deposition of IgGs and complement, and lesion staging by CD68 staining for macrophages in NMO and MS patient samples. Second, we attempted to identify whether there was a correlation between Cx43 astrocyte pathology, oligodendrocyte pathology and clinical and immunological characteristics in MS and NMO, using immunohistochemical methods, clinical evaluation and antibody assay to Cx43.

## Materials and Methods

### Ethics Statement

This study was approved by the ethics committee of Kyushu University Hospital. Informed, written consent from each donor or next of kin was obtained for use of autopsied tissues or blood samples in this research study.

### Autopsy tissue and patient characterization

This study was performed on archival autopsied brain, optic nerve, and spinal cord tissues from 10 NMO cases, including one anti-AQP4 antibody-positive case, one case with NMO spectrum disorder (NMOSD), and six cases with MS, including one with Marburg’s variant who was seronegative for anti-AQP4 antibody. All cases were obtained from the Department of Neuropathology, Kyushu University, with the exception of the Marburg’s variant MS case from Hamamatsu Medical University and the anti-AQP4 antibody-positive NMO case from Tenri Hospital. NMO/NMOSD diagnosis was based on Wingerchuk’s criteria [27–29], and MS was diagnosed according to the Poser criteria [30]. The clinical findings are summarized in Table 1. The median age at autopsy was 44.0 (range 28–88) years in NMO/NMOSD cases (9 females and 2 males), and 37.0 (range 12–52) years old in MS cases (4 females and 2 males). Disease durations ranged from 0.4 to 17.0 years in the NMO/NMOSD group (median 4.7 years), and from 0.3 to 21.0 years in the MS group (median 2.2 years). All patients with MS and NMO/NMOSD died of respiratory diseases except for MS-5 whose cause of death was unknown. All patients suffered from severe paraplegia, quadriplegia or bulbar palsy prior to death, which might be a background cause of death. In addition, we used the same set of control cases with other neurological diseases as in our previous study [22] consisting of myasthenia gravis (MG) (n=1), spastic paraplegia (SPG) type 2 (n=1), amyotrophic lateral sclerosis (ALS) (n=5), encephalitis (n=2, including one positive for anti-N-methyl-D-aspartate receptor antibody and another positive for anti-thyroglobulin antibody), spinocerebellar degeneration (SCD) (n=2), vasculitis (n=3), bacterial meningoencephalitis (n=1), cerebral infarction (n=1), cerebral hemorrhage (n=1), Pick’s disease (n=1), progressive supranuclear palsy (n=3), and multiple system atrophy (n=2).

**Table 1:** Summary of clinical and pathological findings of Japanese patients with NMO and MS.

**Autopsy**	**Age (yrs)**	**Sex**	**Disease** **duration (yrs)**	**Annualized** **relapse rate**	**Clinically estimated** **sites of lesions**	**Pathologically determined** **sites of lesions**	**Cause of death**	**Neurological symptom possibly associated with death**
NMO-1	44	F	3.8	1.6	O2, S6	O, S, Bs, Cr, Cl	Pneumonia	Quadriplegia
NMO-2	44	F	1.8	2.8	O2, Bs3, S2	O, S, Bs, Cr	Pneumonia	Bulbar palsy
NMO-3	48	F	0.5	4.0	O2, Bs1, S1	O, S, Bs, Cr	Aspiration pneumonia	Quadriplegia
NMO-4	32	M	6.3	1.1	O1, Bs2, S7	O, S, Bs, Cl, Cr	Bronchial pneumonia	Quadriplegia, Bulbar palsy
NMO-5	28	F	4.7	0.6	O3, Bs2, S2	O, S, Bs, Cl, Cr	Bronchial pneumonia	Paraplegia, Bulbar palsy
NMO-6	35	F	7.0	1.4	O4, Bs4, S4	O, S, Bs, Cr	Respiratory failure	Paraplegia, Bulbar palsy
NMO-7	37	F	10.8	1.5	O9, Bs2, S14	O, S, Bs, Cl, Cr	Respiratory failure	Quadriplegia, Bulbar palsy
NMO-8	47	F	8.3	0.1	O2, Bs1, S2	O, S, Bs, Cr	Bronchial pneumonia	Bulbar palsy
NMO-9	54	F	4.0	1.3	O1, S6	O, S	Respiratory failure	Bulbar palsy
NMO-10*	37	F	17.0	1.1	O8, Bs1, S9, Cr3	Bs, S, Cr	Sudden death (suspected acute respiratory failure)	Quadriplegia
NMOSD	88	M	0.4	0.0	S1	O, S	Pneumonia	Paraplegia
MS-1	12	F	5.0	2.2	O6, Bs1, S2, Cr3	O, Bs, S, Cr, Cl	Respiratory failure	Quadriplegia, Bulbar palsy
MS-2	35	M	3.1	1.0	O1, Bs2, S2, Cr2	O, Bs, S, Cr	Aspiration pneumonia	Quadriplegia
MS-3	52	F	1.3	1.5	Bs1, Cr3	Bs, Cr	Bronchial pneumonia	Quadriplegia, Bulbar palsy
MS-4	45	F	0.7	1.4	Bs2, S2	O, S, Bs	Pneumonia	Paraplegia
MS-5	39	M	21.0	0.5	O1, Bsx, Clx, Crx	O, Bs, Cl, Cr	Unknown	Bulbar palsy
MS-6	29	F	0.3	4.0	Bs1, Cr3	S, Bs, Cr	Respiratory failure	Quadriplegia

F = female, M = male, MS = multiple sclerosis, NMO = neuromyelitis optica [27,29], NMOSD = neuromyelitis optica spectrum disorder [28].

Lesion sites in clinical exacerbations: O = optic nerve, S = spinal cord, Bs = brainstem, Cl = cerebellum, Cr = cerebrum. Numbers indicate exacerbations in each lesion site (for example, O2 represents two episodes of optic neuritis). x indicates unspecified numbers. Asterisk indicates an NMO case with anti-AQP4 antibody seropositivity.

### Tissue preparation and immunohistochemistry

Autopsy specimens were fixed in 10% buffered formalin and processed into paraffin sections (5-µm thick). The sections were routinely subjected to hematoxylin and eosin (H&E), Klüver-Barrera (KB), and Bodian or Bielschowsky silver impregnation stains. The primary antibodies used for immunohistochemistry are listed in Table 2. AQP4, Cx43, Cx30, glial fibrillary acidic protein (GFAP) and megalencephalic leukoencephalopathy with subcortical cyst 1 (MLC1) were used as astrocyte markers. Cx32, Cx47, oligodendrocyte-specific protein (OSP), myelin-associated glycoprotein (MAG), myelin basic protein (MBP), myelin oligodendrocyte glycoprotein (MOG) and Nogo-A [31] were used as oligodendrocyte/myelin markers, and CD68 was used as a macrophage marker. All sections were deparaffinized in xylene and rehydrated through an ethanol gradient. Endogenous peroxidase activity was blocked with 0.3% (v/v) H_2_O_2_/methanol. Sections were then incubated with primary antibodies at 4°C overnight. After rinsing, sections were subjected to either a streptavidin-biotin complex or an enhanced indirect immunoperoxidase method using Envision (DakoCytomation, Glostrup, Denmark). Immunoreactivity was detected using 3, 3'-diaminobenzidine and sections were counterstained with hematoxylin.

**Table 2:** Antibodies used for immunohistochemistry.

**Antigen**	**Type**	**Dilution**	**Antigen retrieval**	**Source**
Astrocyte				
Cx43	rabbit polyclonal	1:1000	N.D.	Abcam, Cambridge, UK
Cx30	rabbit polyclonal	1:100	N.D.	Sigma Aldrich, St Louis, USA
AQP4	rabbit polyclonal	1:500	N.D.	Santa Cruz Biotechnology, California, USA
GFAP	rabbit polyclonal	1:1000	N.D.	DakoCytomation, Glostrup, Denmark
GFAP	mouse monoclonal	1:100	Autoclave/10 mM citrate buffer	DakoCytomation, Glostrup, Denmark
MLC1	rabbit polyclonal	1:400	N.D.	Sigma Aldrich, St Louis, USA
Oligodendrocyte/myelin				
Cx32	rabbit polyclonal	1:50	N.D.	Abcam, Cambridge, UK
Cx47	rabbit polyclonal	1:100	N.D.	Abcam, Cambridge, UK
OSP	rabbit polyclonal	1:1000	N.D.	Abcam, Cambridge, UK
MAG	rabbit polyclonal	1:400	N.D.	Sigma Aldrich, St Louis, USA
MOG	rabbit polyclonal	1:1000	N.D.	Sigma Aldrich, St Louis, USA
MBP	mouse monoclonal	1:50	Autoclave/10 mM citrate buffer	Novocastra, Newcastle upon Tyne, UK
Nogo-A	rabbit polyclonal	1:400	N.D.	Santa Cruz Biotechnology, California, USA
Macrophage				
CD68	mouse monoclonal	1:200	Autoclave/10 mM citrate buffer	DakoCytomation, Glostrup, Denmark
Axon				
Neurofilament	mouse monoclonal	1:100	N.D.	DakoCytomation, Glostrup, Denmark
Complement				
C3d	rabbit polyclonal	1:1000	Autoclave/10 mM citrate buffer	DakoCytomation, Glostrup, Denmark
C9neo	mouse monoclonal	1:1000	Autoclave/10 mM citrate buffer	Abcam, Cambridge, UK
Immunoglobulin				
IgG	rabbit polyclonal	1:10,000	Autoclave/10 mM citrate buffer	DakoCytomation, Glostrup, Denmark
IgM	rabbit polyclonal	1:10,000	Autoclave/10 mM citrate buffer	DakoCytomation, Glostrup, Denmark
T cell				
CD45RO	mouse monoclonal	1:200	Autoclave/10 mM citrate buffer	DakoCytomation, Glostrup, Denmark

N.D. = not done, AQP4 = aquaporin-4, Cx = connexin, GFAP = glial fibrillary acidic protein, MAG = myelin-associated glycoprotein, MBP = myelin basic protein, MLC1 = megalencephalic leukoencephalopathy with subcortical cyst 1, MOG = myelin-oligodendrocyte glycoprotein, OSP = oligodendrocyte-specific protein

### Indirect immunofluorescence and confocal laser microscopy

Using the same set of paraffin sections described above, double immunofluorescence staining was performed with the following combinations of antibodies: mouse monoclonal anti-human Cx43 and rabbit polyclonal anti-human Cx47; mouse monoclonal anti-human GFAP and rabbit polyclonal anti-human Cx32; and, mouse monoclonal anti-human GFAP and rabbit polyclonal anti-human Cx47. Sections were deparaffinized in xylene and rehydrated through an ethanol gradient. Sections were then incubated with primary antibodies overnight at 4°C. After rinsing, sections were incubated with Alexa 488-conjugated goat anti-rabbit IgG or Alexa 546-conjugated goat anti-mouse IgG (Invitrogen) and then counterstained with DAPI. Images were captured using a confocal laser microscope system (Nikon A1, Nikon, Japan).

### Staging of demyelinating lesions

We classified demyelinating lesions into three stages, i) actively demyelinating lesions, ii) chronic active lesions and iii) chronic inactive lesions, based on the density of macrophages phagocytosing myelin debris [32,33]. Actively demyelinating lesions were destructive lesions densely and diffusely infiltrated with macrophages phagocytosing myelin debris, as identified by Luxol fast blue staining and immunohistochemistry for minor myelin proteins (MAG, OSP and MOG). Chronic active lesions were defined as those with hypercellularity of macrophages restricted to the periphery of lesions. Chronic inactive lesions showed no increase in macrophage numbers throughout the lesions.

### Correlation of Cx expression with myelin loss and astrogliosis

For each lesion, we compared Cx43, Cx30, Cx47 and Cx32 expression levels with the spatial distribution of myelin loss. The expression levels of Cxs from region-matched unaffected white matter in the same section were used as internal controls. We confirmed the existence of astrocytes by GFAP and MLC1 [34] staining in neighboring sections for all lesions. This allowed us to exclude secondary down-modulation of Cx43 resulting from the loss of astrocytes in necrotic lesions. We also strictly evaluated Cx43 expression status in preserved astrocytes in and around the lesions, and compared the expression levels and distribution of Cx43 with those of AQP4 in all lesions. Using serial sections of demyelinating lesions, we classified the expression patterns of individual connexin (Cx43, Cx47 and Cx32) immunoreactivity relative to the intensity of GFAP immunoreactivity and myelin staining into the following five patterns, as previously described [16] (Table 3): pattern A (area of diminished Cx immunoreactivity extending beyond the area of myelin loss), pattern B (area of diminished Cx immunoreactivity conforming with the area of myelin loss), pattern C (area of diminished Cx immunoreactivity is smaller than the area of myelin loss), pattern D (preserved Cx immunoreactivity with loss of myelin staining) and pattern N (necrosis or cavity formation; GFAP-negative, Cx-negative and myelin-negative by definition). Patterns associated with focal necrosis or cavity formation were expressed as “patterns X & N” (X = A, B, C or D).

**Table 3:** Classification of demyelinating lesions according to Cx43, Cx47, Cx32, GFAP and myelin protein (MOG, MBP and OSP) immunostaining and Klüver-Barrera staining.

**Lesion pattern**	**Extents of Cx and myelin loss**	**GFAP expression**
Pattern A	Cx loss or decrease > myelin loss	(+)
Pattern B	Cx loss or decrease = myelin loss	(+)
Pattern C	Cx loss or decrease < myelin loss	(+)
Pattern D	Cx totally preserved in areas of myelin loss	(+)
Pattern N	Necrosis or cavity formation	(-)

Cx = connexin, GFAP = glial fibrillary acidic protein, MBP = myelin basic protein, MOG = myelin-oligodendrocyte glycoprotein, OSP = oligodendrocyte-specific protein

### Anti-Cx43 antibody assay

Anti-Cx43 antibody levels were measured by immunofluorescence using Cx43-GFP fusion protein-transfected HEK-293 cells, as described previously [22]. Personnel conducting the assays were blinded to the specimen origin, and assays were performed at least twice for each sample. Samples that yielded a positive result twice were designated ‘positive’. Sera from 120 subjects including 30 MS, 30 NMO, 20 atopic myelitis (AM), 20 other neurological disorders and 20 healthy controls were examined.

### Statistical Analyses

Statistical analyses of disease duration and annualized relapse rates were performed using the Mann–Whitney *U*-test. Differences in death frequency within two years after the disease onset and relationships between inflammatory components and Cx43 status were tested for significance using Fisher’s exact probability test.

## Results

### Immunohistochemical findings in control cases

Staining patterns of astrocytic Cx43 and Cx30 and oligodendrocytic Cx47 and Cx32 in normal and diseased CNS tissues were described in detail in our previous report [22]. Briefly, in normal cerebral tissues, Cx43 and Cx30 staining was more pronounced in the cortex than in white matter, with stronger staining in the perivascular foot processes. Cx30 is insignificantly expressed in normal white matter. Glial limiting membranes and subependymal astrocytes strongly expressed Cx43 and Cx30. In contrast, GFAP immunoreactivity was preferentially observed in the cerebral white matter whereas in the cortex, except for strong staining of glial limiting membranes, only a few astrocytes were immunopositive for GFAP. Reactive astrocytes and gliotic scars were strongly immunopositive for both Cx43 and GFAP. Cx32, Cx47 and Cx43 were also abundantly expressed in the optic nerve (Figure S1). High power magnification of cross sections of the optic nerve revealed expression of Cx32 and Cx47 along myelin sheaths and on the surface of oligodendrocytes adjacent to the myelin sheaths (Figure S1A and C). Longitudinal sections of the optic nerve showed expression of Cx32 and Cx47 around the interfascicular oligodendrocyte chain (Figure S1B and D). Immunoreactivity of Cx43 was observed in astrocytes and astrocyte perivascular foot processes (Figure S1E and F).

### Immunohistochemical findings in NMO and NMOSD cases

#### Astrocytic Cx43 and Cx30 in NMO and NMOSD lesions

Five NMO cases (NMO-2, 3, 4, 7 and 10) and one NMOSD case showed loss of Cx43 and AQP4 staining in degenerative GFAP-positive astrocytes in active lesions beyond the demyelinated areas as determined by myelin protein (MOG, MBP, and OSP) staining. The remaining five NMO cases (NMO-1, 5, 6, 8 and 9) showed preservation of Cx43 and AQP4 in GFAP-positive astrocytes in active lesions (Table 4). The frequency of each Cx43 expression pattern in NMO/NMOSD lesions is summarized in Table 5. In actively demyelinating lesions, 26 of 35 (74.3%) lesions were classified as pattern A or B, while 18 of 41 (43.9%) chronic active lesions were classed as pattern A or B. None of the 13 chronic inactive lesions was classed as pattern A or B. Cx30 expression levels were very low in astrocytes in NMO/NMOSD lesions, similar to that in the white matter of normal controls (data not shown).

**Table 4:** Summary of connexin 43 immunoreactivity patterns in demyelinating lesions from cases with NMO and NMO spectrum disorder.

**Autopsy**	**Stage**	**Pattern**				
		**Cerebrum**	**Brainstem**	**Cerebellum**	**Spinal cord**	**Optic nerve**
Preferential Cx43 loss or decrease						
NMO-2	Active	A (1)	A&N (1), B (1)	N.A.	B&N (1)	N.A.
	Chronic active	B&N (2)			A&N (1), B&N (1)	
NMO-3	Active					A&N (1)
	Chronic active	B&N (1), C&N (1)	A (1)		A&N (4)	A&N (1)
NMO-4	Active	A (1), B (1)	A (2), B (2)		B&N (2)	B (1), C (1)
	Chronic active		B (1)		C&N (1)	
NMO-7	Active		A (3), C (1)		A&N (5)	
	Chronic active		B (1)			
	Chronic inactive					D&N (1)
NMO-10	Active	A (1), A&N (1)				N.A.
	Chronic active	B (2), B&N (1)	C&N (1)		B&N (1), C&N (1)	
	Chronic inactive	C (1)				
NMOSD	Active				A&N (1), B&N (1), N (1)	
	Chronic active				B&N (1)	
	Chronic inactive					D (1)
Preserved Cx43 expression						
NMO-1	Chronic active		D (1)		D (1)	D (1)
	Chronic inactive					D (1)
NMO-5	Active	D (1)				
	Chronic active	D (1)	D (2)	D (1)		
	Chronic inactive		D&N (1)		D&N (1)	D (1)
NMO-6	Active	D (1)				
	Chronic active	D (4)	D&N (1)			
	Chronic inactive		D&N (1)		D (1), D&N (1)	D&N (1)
NMO-8	Chronic active	D (2), D&N (2)	D&N (1)			D (1)
	Chronic inactive		D (1)			N (1)
NMO-9	Active				D&N (1), N (2)	D (1)
	Chronic active				D&N (1)	

Blank cell = no lesions, Cx43 = connexin 43, NMO = neuromyelitis optica, NMOSD = neuromyelitis optica spectrum disorder, N.A. = specimen not available. Patterns associated with focal necrosis or cavity formation were expressed as “patterns X & N” (X = A, B, C or D). Patterns are described in Table 3 and Methods.

**Table 5:** Frequency of Cx43, Cx47 and Cx32 immunoreactivity patterns in demyelinating lesions from cases with NMO and NMO spectrum disorder.

	**NMO and NMOSD (n=89)**				**NMO and NMOSD (n=89)**		
**Cx43 expression pattern**	**Active (n=35)**	**Chronic active (n=41)**	**Chronic inactive (n=13)**	**Cx47 and Cx32 expression pattern**	**Active (n=35)**	**Chronic active (n=41)**	**Chronic inactive (n=13)**
Pattern A	17	7	0	Pattern A	0	13	5
Pattern B	9	11	0	Pattern B	32	28	7
Pattern C	2	4	1	Pattern C	0	0	0
Pattern D	4	19	11	Pattern D	0	0	0
Pattern N	3	0	1	Pattern N	3	0	1

See Table 3 for the pattern definitions. Any pattern with necrosis (pattern X & N) was included as pattern X.

Cx = connexin, MS = multiple sclerosis, NMO = neuromyelitis optica, NMOSD = neuromyelitis optica spectrum disorder

#### Loss of AQP4 and Cx43 extending beyond demyelination in active perivascular lesions

**Figure 1:**
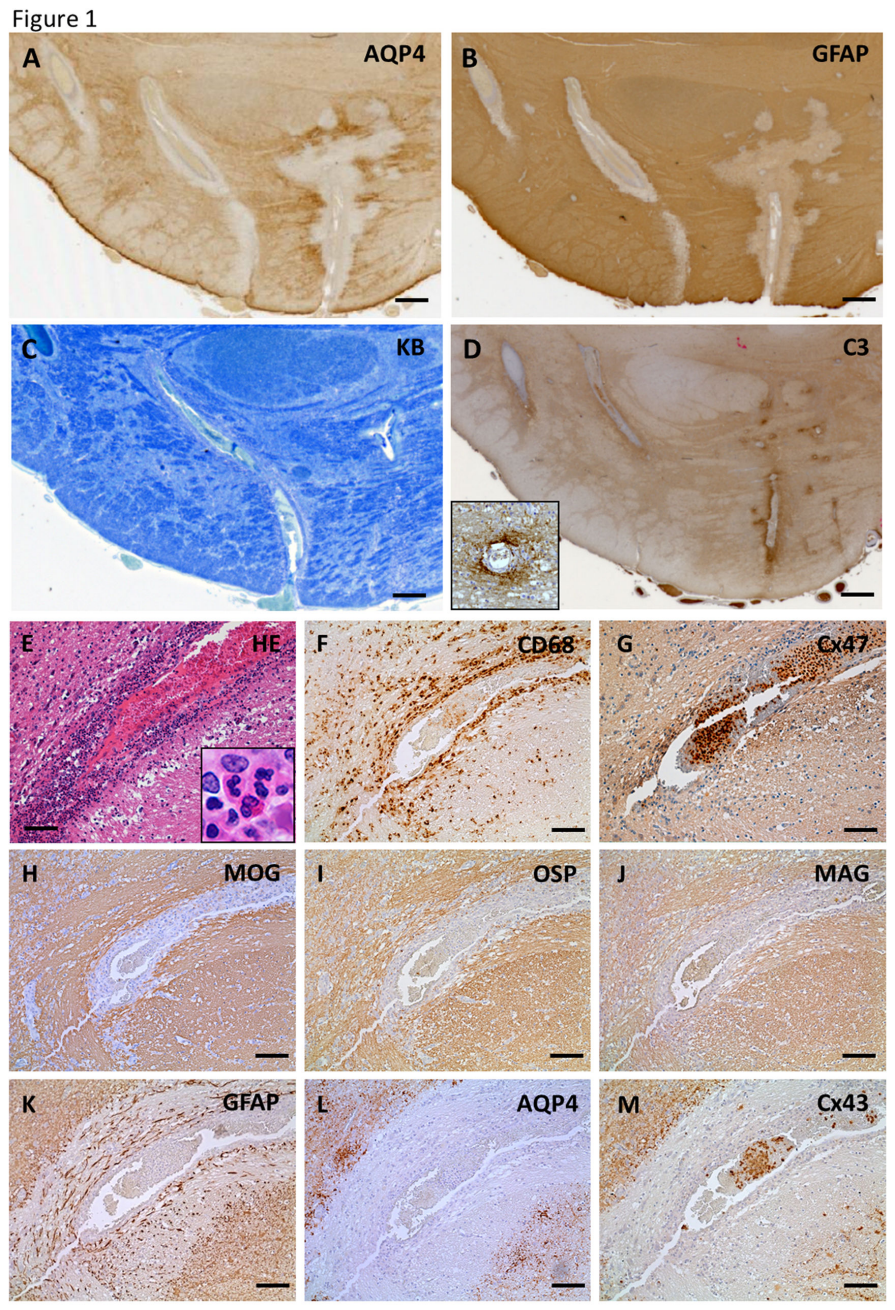
Loss of AQP4 and Cx43 in perivascular lesions of neuromyelitis optica (NMO) in case NMO-4 (A–M) Immunoreactivities for AQP4 and GFAP are markedly diminished around blood vessels in the ventral side of the pons (A, B). In contrast, myelin is preserved around the same blood vessels in the perivascular areas as assessed by KB staining (C). Complement components are specifically deposited in the affected perivascular spaces (D, insert). Serial sections of affected blood vessel from the same case (E–M). Perivascular inflammatory infiltrations contain a large number of neutrophils, mononuclear cells (inset to E), CD68-positive macrophages (E, F) and few eosinophils. Myelin proteins, including Cx47 (G), MOG (H), OSP (I) and MAG (J), are preserved around the vessels. AQP4 and Cx43 are completely absent in highly degenerative, GFAP-positive astrocytes (K–M). This lesion is classified as pattern A for Cx43 and pattern B for Cx47/Cx32. Scale Bar = 0.4 mm (A–D); 100 µm (E–M).

Five NMO cases (NMO-2, 3, 4, 7 and 10) and one NMOSD case showed loss of Cx43 in degenerative GFAP-positive astrocytes beyond the demyelinated areas in at least one active demyelinating lesion (pattern A) (Figure 1, Table 4). Perivascular deposition of complement and immunoglobulin were observed around affected blood vessels in 19 lesions from four NMO cases (NMO-2, 4, 7 and 10). Case NMO-4 showed typical perivascular astrocytopathy with complement deposition around the pontine blood vessels, where preserved myelin was revealed by KB staining (Figure 1A–D). Perivascular inflammatory infiltration consisted of a large number of neutrophils and mononuclear cells including macrophages and low numbers of eosinophils (Figure 1E). No CD68-positive macrophages phagocytosing myelin debris were observed around blood vessels in the lesions (Figure 1F). All myelin proteins including MAG, OSP, MOG and MBP as well as Cx32 and Cx47 were relatively preserved around blood vessels (Figure 1G–J). However, immunoreactivity for AQP4 and Cx43 was completely absent in highly degenerative, GFAP-positive astrocytes (Figure 1K–M). Double immunostaining for oligodendrocytic Cxs and GFAP also revealed the relative preservation of Cx32 and Cx47 in perivascular regions containing markedly degenerated astrocytes (Figure S2).

#### Oligodendrocytic Cx32 and Cx47 in NMO and NMOSD lesions

Low expression of oligodendrocytic Cx32 and Cx47 and myelin loss determined by myelin protein (MOG, MBP, and OSP) staining was observed in all NMO/NMOSD cases. The frequency of Cx32 and Cx47 expression patterns in NMO/NMOSD lesions is summarized in Table 5. Pattern A or B lesions were observed in 32 of 35 (91.4%) actively demyelinating lesions, in all 41 (100%) chronic active lesions, and 12 of 13 (92.3%) chronic inactive lesions. Thus, all NMO/NMOSD cases had pattern A or B lesions in terms of oligodendrocytic Cx32 and Cx47 expression.

#### Loss of Cx32 and Cx47 extending beyond the demyelinated area in chronic lesions

**Figure 2:**
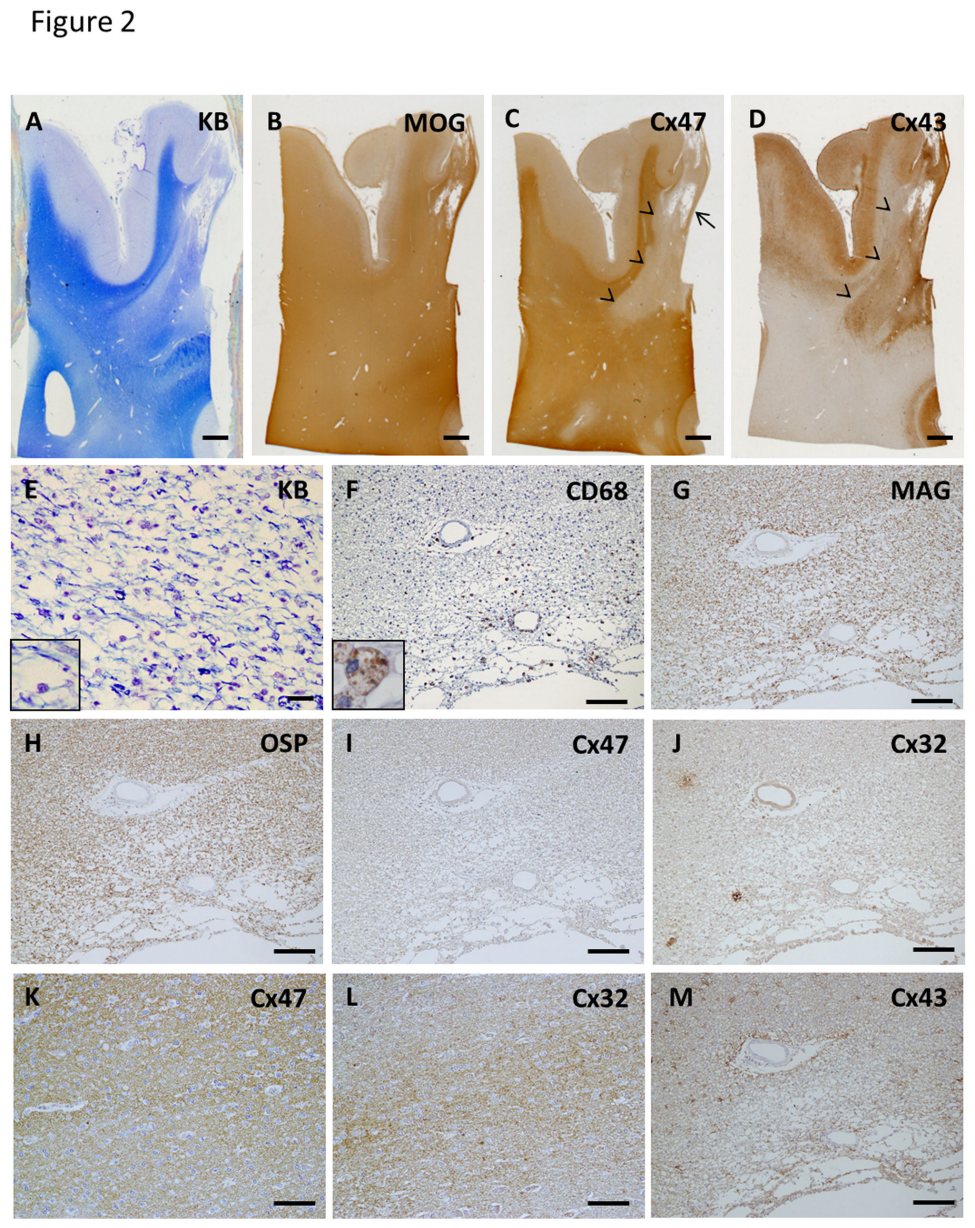
Loss of Cx47 and Cx32 in chronic active NMO lesions in case NMO-6 (A–M) Low magnification view of KB staining (A) and immunostainings for MOG (B), Cx47 (C) and Cx43 (D) in the cerebrum. Immunoreactivity for Cx47 is extensively diminished in the demyelinating lesion with cavitation in the cerebral white matter, whereas immunoreactivity for MOG is relatively preserved (A–C). This lesion is classified as pattern D for Cx43 and pattern A for Cx47/Cx32. Up-regulation of Cx43 immunoreactivity is observed in the corresponding demyelinating area (D). Arrows indicates cavitation and arrowheads show the lesion boundary (C, D). CD68-positive foamy macrophages are observed at the periphery and perivascular areas of the lesion. No foamy macrophages contain KB-positive granules within their cytoplasm (E, F, insert). Immunoreactivities for MAG and OSP are decreased in these lesions (G, H). Expression levels of Cx47 and Cx32 are extensively reduced in the lesion (I, J) compared with the non-affected white matter (K, L). Numerous Cx43- positive astrocytes are present in this lesion suggesting astrogliosis. Cx43 immunoreactivity is also preserved in the perivascular areas (M). Scale Bar = 2 mm (A–D); 20 µm (E); 100 µm (F–J, M); 50 µm (K, L).

In actively demyelinating lesions, none of 35 lesions was classified as pattern A. In contrast, 13 of 41 (31.7%) chronic active lesions showed pattern A and five of 13 (38.4%) chronic inactive lesions were classed as pattern A. Five NMO cases (NMO-3, 5, 6, 7 and 10) showed loss of Cx32 and Cx47 beyond the demyelinated areas in at least one chronic active or chronic inactive lesion (pattern A) (Figure 2). In a chronic active cerebral white matter lesion from case NMO-6, immunoreactivity for Cx47 was diminished beyond the demyelinated area revealed by immunostaining for MOG (Figure 2A–C). Immunoreactivity for Cx43 was increased conforming to the demyelinating lesion (Figure 2D). CD68-positive foamy macrophages infiltrated at the periphery and perivascular areas of the lesion while KB staining revealed the foamy macrophages did not contain KB-positive granules within their cytoplasm (Figure 2E, F). Immunoreactivity for MAG and OSP was diminished in the lesion while none of the foamy macrophages had phagocytosed myelin debris, as they were immunonegative for these myelin proteins (Figure 2G, H). Immunoreactivity for Cx47 and Cx32 was extensively diminished in the lesion compared with non-affected white matter (Figure 2I–L). In contrast, immunoreactivity for Cx43 was up-regulated because of astrogliosis (Figure 2M).

#### Pronounced loss of MAG and apoptotic oligodendrocytes in parenchymal lesions of NMO

**Figure 3:**
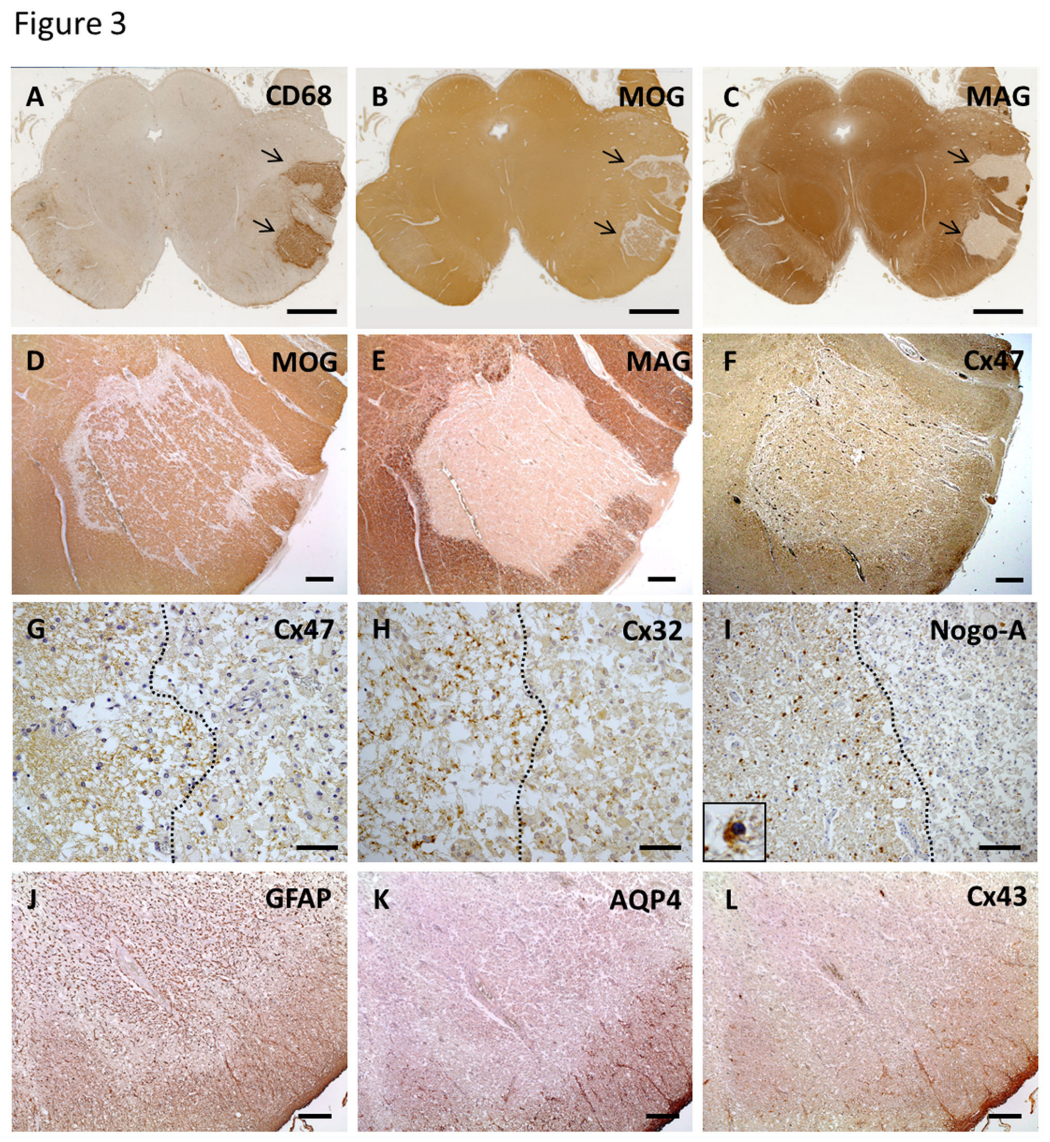
Coexistence of distal oligodendrogliopathy in active NMO lesions in case NMO-4 (A–L) CD68 immunostaining demonstrates massive infiltration of macrophages in the cerebral peduncle (A, arrows). Immunoreactivity for MOG is relatively preserved but is completely lost for MAG (B, C, arrows). Higher magnification of the lesion (D–L). Immunoreactivity for MOG is relatively preserved in contrast to complete loss of MAG in this lesion (D, E). Cx47 expression is diminished compared with non-affected white matter (F). Lesion boundary areas (G–I). A dotted line indicates the boundary. Immunostainings for Cx47 and Cx32 are slightly diminished but preserved inside the lesion (G, H). Immunostaining of Nogo-A is markedly decreased and apoptotic nuclear condensation of oligodendrocytes is present (I, insert). Immunoreactivities for AQP4 and Cx43 within highly degenerative GFAP-positive astrocytes are completely lost (J–L). This lesion is classified as pattern B for Cx43 and pattern B for Cx47/Cx32. Scale Bar = 4 mm(A-C); 0.5 mm (D–F); 50 µm (G, H); 100 µm (I); 200 µm (J–L).

Eight active lesions from three NMO cases (NMO-2, 4 and 10) showed a pronounced loss of MAG compared with other myelin proteins and apoptotic oligodendrocytes with typical NMO features. The NMO-4 case showed three parenchymal lesions in the brainstem white matter. In active lesions of the cerebral peduncle with massive macrophage infiltration, immunoreactivity for MAG was completely absent, while that of MBP was relatively preserved (Figure 3A–C, E). Immunoreactivities for other myelin proteins, such as MOG, were also preserved (Figure 3D). Cx32 and Cx47 expression were diminished compared with non-affected white matter. However, upon higher magnification, punctate immunoreactivity for these Cxs was still observed (Figure 3F–H). Immunostaining of Nogo-A was profoundly decreased and residual oligodendrocytes showed apoptotic nuclear changes (Figure 3I). Immunoreactivity for AQP4 and Cx43 in highly degenerative GFAP-positive astrocytes was completely absent (Figure 3J–L).

#### Pattern of demyelination and astrocytopathy in an anti-AQP4 antibody-positive NMO case

**Figure 4:**
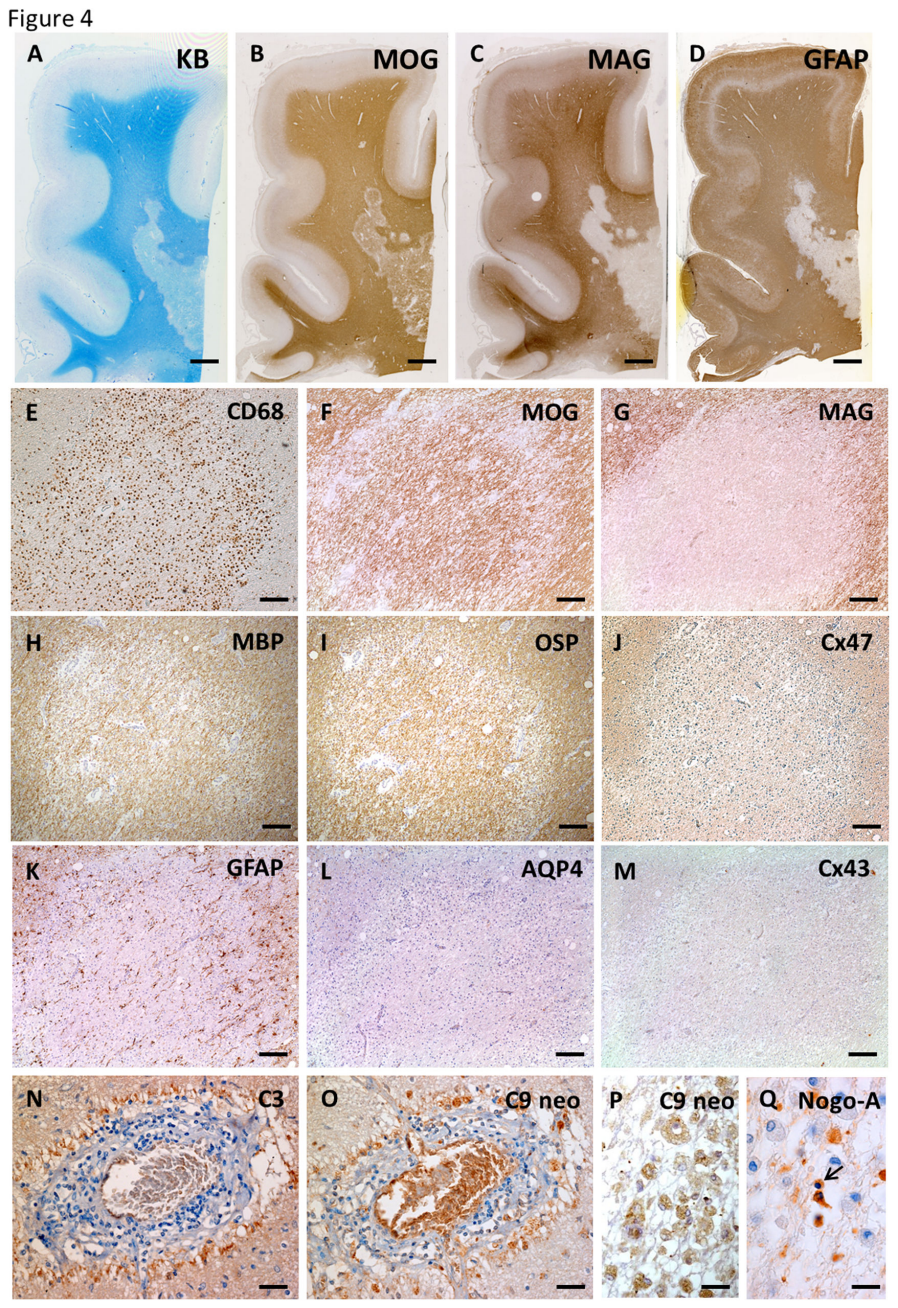
Distal oligodendrogliopathy and astrocytopathy in anti-AQP4 antibody-seropositive NMO (case NMO-10). In active lesions of the cerebral white matter, KB staining and MOG immunostaining show remaining myelin (A, B). Patterns of preferential MAG loss and marked loss of GFAP immunoreactivity are seen (C, D). Higher magnification reveals sharply demarcated, prominent MAG loss in this lesion with infiltration of numerous CD68-positive macrophages, whereas immunoreactivity for MOG, MBP and OSP is preserved in the lesion (E–I). Immunoreactivity for Cx47 is diminished compared with non-affected white matter (J). Complete loss of AQP4 and Cx43 in degenerative, GFAP-positive astrocytes (K–M) and complement deposition are observed around blood vessels with perivascular cell cuffing (N, O). Complement components are present within foamy macrophages in this lesion (P). Nogo-A-positive oligodendrocytes are markedly decreased in this lesion and some remaining oligodendrocytes show nuclear condensation, suggesting apoptotic changes (Q). This lesion is classified as pattern A for Cx43 and pattern B for Cx47/Cx32. Scale Bar = 4 mm (A–D); 200 µm (E–M); 50 µm (N, O); 20 µm (P, Q).

The NMO-10 case was seropositive for anti-AQP4 antibody. In active lesions of the cerebral white matter, a demyelination pattern of preferential MAG loss and marked GFAP loss was also observed (Figure 4A–D). Higher magnification revealed sharply demarcated, prominent MAG loss in the lesion with infiltration of numerous CD68-positive foamy macrophages (Figure 4E–G). MBP and OSP were preserved in the lesion, whereas Cx47 expression was diminished (Figure 4H–J). There was a complete absence of AQP4 and Cx43 expression in degenerative, GFAP-positive astrocytes while complement and immunoglobulin deposition was observed around the blood vessels (Figure 4K–O). Immunopositivity of complement components was also observed within numerous infiltrating macrophages (Figure 4P). Nogo-A-positive oligodendrocytes were markedly decreased in this lesion and some residual oligodendrocytes showed nuclear condensation suggesting apoptotic changes (Figure 4Q).

### Immunohistochemical findings in MS cases

#### Astrocytic Cx43 and Cx30 in MS lesions

Three MS cases (MS-3, 4, and 6) showed Cx43, AQP4, and MLC1 loss in GFAP-positive astrocytes in active lesions beyond the areas of demyelination as determined by myelin protein staining, whereas the remaining three MS cases (MS-1, 2, and 5) showed preservation of astrocytic proteins including Cx43, AQP4, MLC1 and GFAP in actively demyelinating lesions (Table 6). The frequency of each Cx43 expression pattern in MS lesions is summarized in Table 7. In actively demyelinating lesions, 15 of 24 (62.5%) lesions were classified as pattern A or B, while seven of 17 (41.2%) chronic active lesions showed pattern A or B. None of 10 chronic inactive lesions had pattern A or B. Cx30 expression levels were very low in the astrocytes of MS lesions, similar to levels in the white matter of normal controls (data not shown).

**Table 6:** Summary of Cx43 immunoreactivity patterns in demyelinating lesions in cases with MS.

**Autopsy**	**Stage**	**Pattern**				
		**Cerebrum**	**Brainstem**	**Cerebellum**	**Spinal cord**	**Optic nerve**
Preferential Cx43 loss or decrease						
MS-3	Active	B (1)	A (1), A&N (2), N (2)			
	Chronic active	B (1)				
	Chronic inactive	C (1)				
MS-4	Active		A (1), B (2)			
	Chronic active		B (1)		A &N (3), A (1)	C (1)
MS-6	Active	A (1), B (2), C (1)	A (1), B (2)		A (1), B (1)	
	Chronic active				B (1)	
Preserved Cx43 expression						
MS-1	Active		N (3)			
	Chronic active	D&N (1)				
	Chronic inactive				D (1), D&N (2)	
MS-2	Active		D&N (1)			
	Chronic active		D (2)			D (2)
MS-5	Active	D (2)				
	Chronic active	D (3)	D (1)			
	Chronic inactive	D (4)		D (1)		D (1)

Blank cell = no lesions, Cx43 = connexin 43, MS = multiple sclerosis. Patterns associated with focal necrosis or cavity formation were expressed as “patterns X & N” (X = A, B, C or D). Patterns are described in Table 3 and Methods.

**Table 7:** Frequency of Cx43, Cx47 and Cx32 immunoreactivity patterns in demyelinating lesions from cases with MS.

	**MS (n=51)**				**MS (n=51)**		
**Cx43 expression pattern**	**Active (n=24)**	**Chronic active (n=17)**	**Chronic inactive (n=10)**	**Cx47 and Cx32 expression pattern**	**Active (n=24)**	**Chronic active (n=17)**	**Chronic inactive (n=10)**
Pattern A	7	4	0	Pattern A	0	2	2
Pattern B	8	3	0	Pattern B	14	13	8
Pattern C	1	1	1	Pattern C	2	2	0
Pattern D	3	9	9	Pattern D	0	0	0
Pattern N	5	0	0	Pattern N	5	0	0

See Table 3 for the pattern definitions. Any pattern with necrosis (pattern X & N) was included as pattern X.

Cx = connexin, MS = multiple sclerosis, NMO = neuromyelitis optica, NMOSD = neuromyelitis optica spectrum disorder

#### Loss of AQP4 and Cx43 staining in actively demyelinating lesions of MS

**Figure 5:**
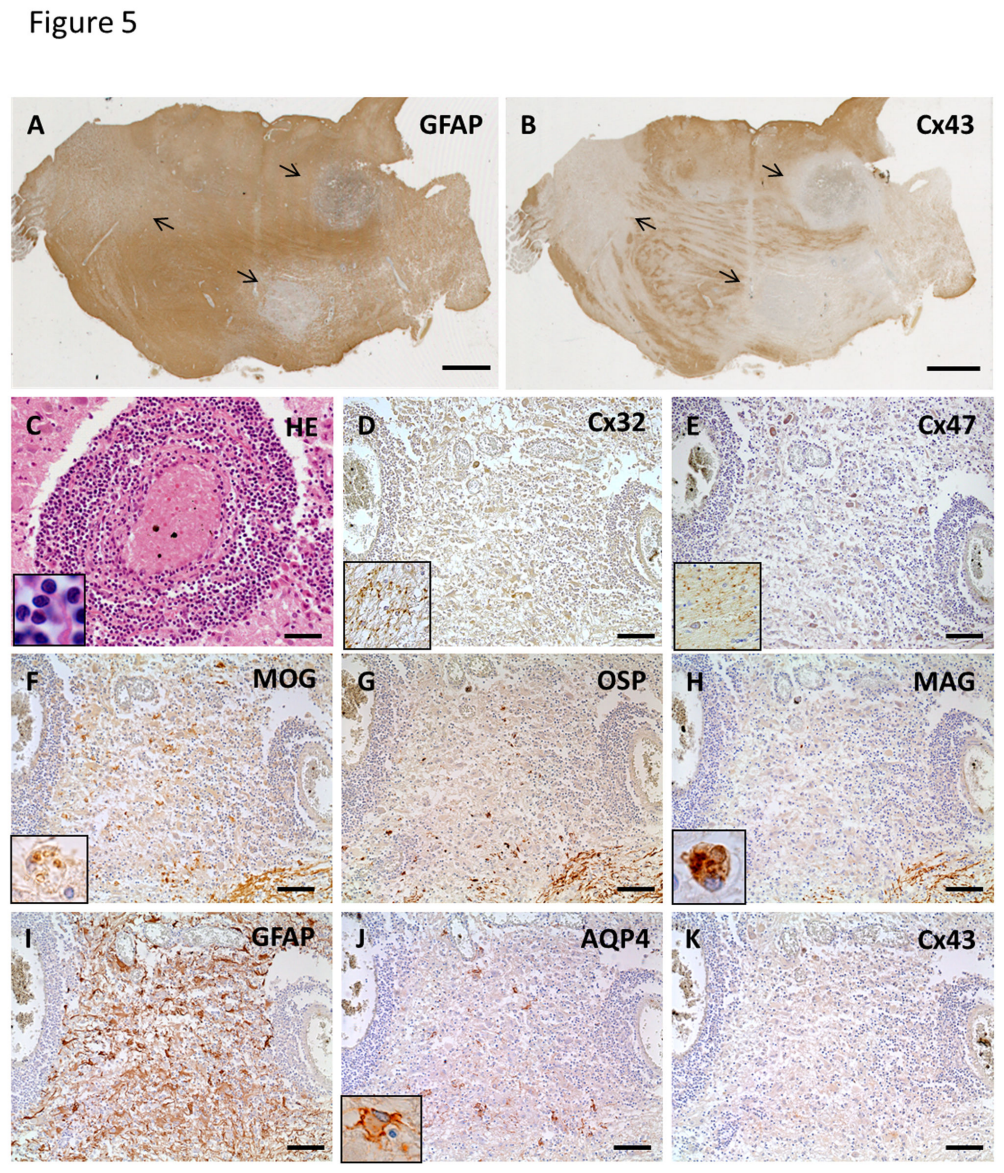
Cx43 and AQP4 astrocytopathy in active lesions of MS (case MS-3). Low magnification view of GFAP (A) and Cx43 (B) immunostaining in the pons. Immunoreactivity for Cx43 is markedly diminished in multiple lesions covered by GFAP-positive astrocytes (A, B, arrows). Massive perivascular cuffing, mostly consisting of lymphocytes, is observed in these lesions (C). Immunoreactivities for Cx32 and Cx47 are decreased in these lesions (D, E) compared with non-affected white matter (D, E, insert). Loss of MAG compared with MOG and OSP is prominent (F–H). Infiltration of macrophages phagocytosing myelin debris, which are immunopositive for myelin proteins (F, H, insert). Numerous GFAP-positive hypertrophic astrocytes exist in perivascular areas and parenchyma of these lesions (I). Patchy loss of AQP4 and diffuse loss of Cx43 in the center and periphery of lesions (J, K). Some hypertrophic astrocytes demonstrate membranous staining for AQP4 (J, insert). This lesion is classified as pattern A for Cx43 and pattern B for Cx47/Cx32. Scale Bar = 4 mm (A, B); 50 µm (C); 100 µm (D–K).

The MS-3 case had cerebral signs at disease onset and brainstem signs at relapse, but neither optic neuritis nor myelitis was noted. Active demyelinating lesions with dense perivascular lymphocytic cuffing existed in the pons, and these lesions showed extensive loss of Cx43 in contrast to GFAP staining (Figure 5A, B). Inflammatory infiltrates constituting perivascular cuffing mostly contained lymphocytes (Figure 5C). Immunoreactivities for Cx32 and Cx47 were markedly decreased in these lesions (Figure 5D, E). Loss of MAG was prominent compared with MOG and OSP, and there was infiltration of macrophages phagocytosing myelin debris, which were immunopositive for myelin proteins (Figure 5F–H). Numerous GFAP-positive reactive astrocytes were preserved in the perivascular areas and parenchyma of these lesions (Figure 5I). Notably, patchy loss of AQP4 and diffuse loss of Cx43 were observed in the center and periphery of lesions (Figure 5J, K).

#### Oligodendrocytic Cx32 and Cx47 in MS lesions

The frequency of Cx32 and Cx47 expression patterns in MS lesions is summarized in Table 7. In actively demyelinating lesions, 17 of 24 (70.8%) lesions were classified as pattern A or B, 15 of 17 (88.0%) chronic active lesions showed pattern A or B and all 10 chronic inactive lesions were pattern A or B. Thus, all MS cases had pattern A or B lesions in terms of oligodendrocytic Cx32 and Cx47 expression.

#### Loss of Cx32 and Cx47 extending beyond the demyelinated area in chronic lesions

**Figure 6:**
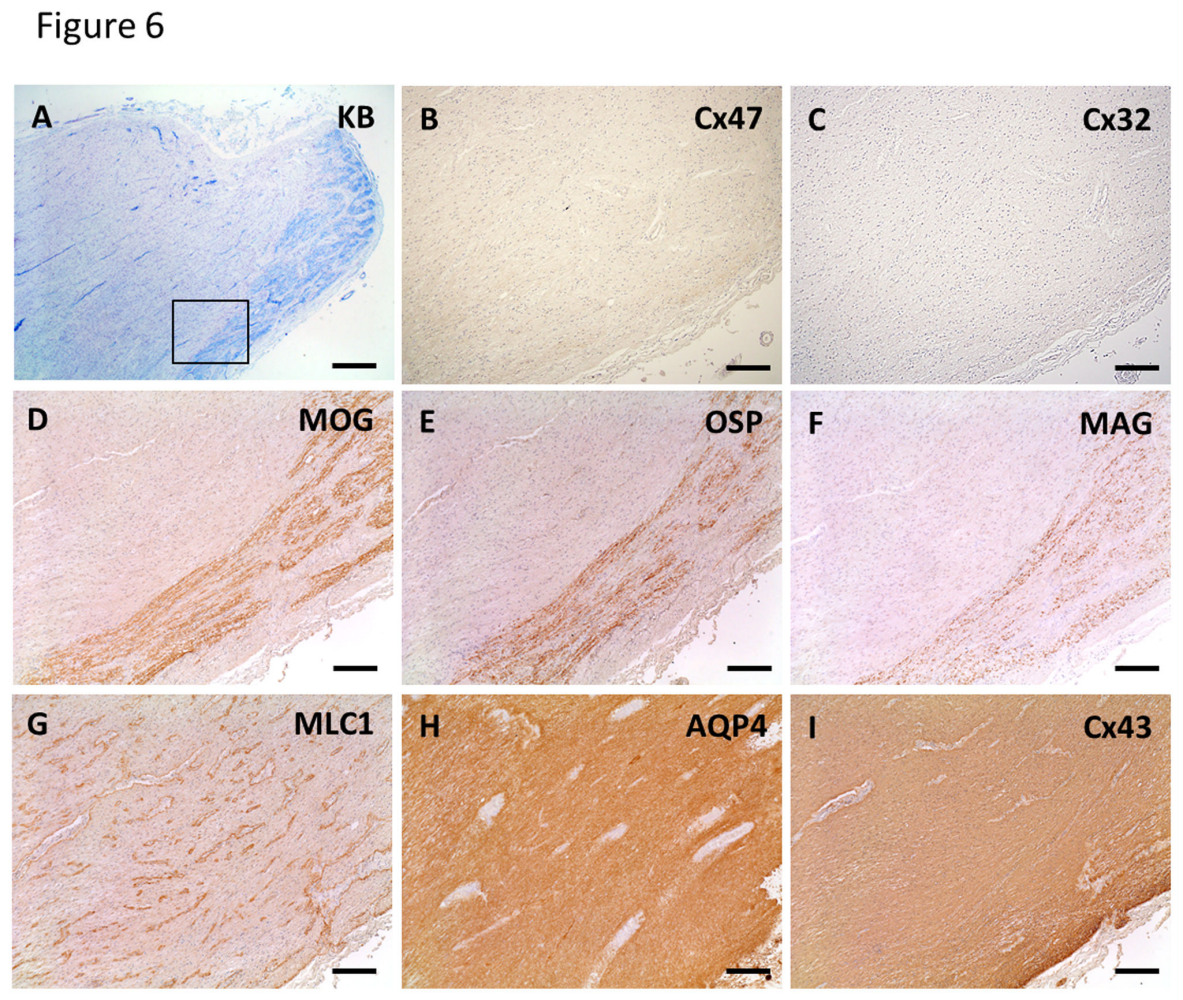
Loss of oligodendrocytic Cx47 and Cx32 expression in chronic lesions of MS (case MS-5). KB staining in a chronic inactive lesion of the optic nerve (A). Higher magnification view of the lesion boundary area (B–I, corresponding to Figure 5A, square). Immunoreactivities for Cx47 and Cx32 are diminished beyond the demyelinated area (B, C) as revealed by immunostaining for MOG, OSP and MAG (D–F). This lesion is classified as pattern D for Cx43 and pattern A for Cx47/Cx32. In contrast, MLC1, AQP4 and Cx43 expression are up-regulated because of astrogliosis (G–I). MLC1 is localized at perivascular foot processes even in chronic gliotic tissues (G). Scale Bar = 1 mm(A); 200 µm (B–I).

One MS case (MS-5) showed loss of Cx32 and Cx47 in lesions beyond the demyelinated areas (pattern A) (Figure 6). In actively demyelinating lesions, none of 24 lesions was classified as pattern A, while two of 17 (11.7%) chronic active lesions and two of 10 (20%) chronic inactive lesions were pattern A. In a chronic inactive optic nerve lesion of MS-5, immunoreactivities for Cx47 and Cx32 were decreased beyond the demyelinated areas, as revealed by KB staining and immunostaining for MOG, OSP and MAG (Figure 6A–F). In contrast, MLC1, AQP4 and Cx43 were up-regulated because of astrogliosis (Figure 6G–I). Notably, MLC1 was localized at perivascular foot processes even in chronic gliotic tissues (Figure 6G).

#### Neuropathological features of an acute Marburg’s type MS case

**Figure 7:**
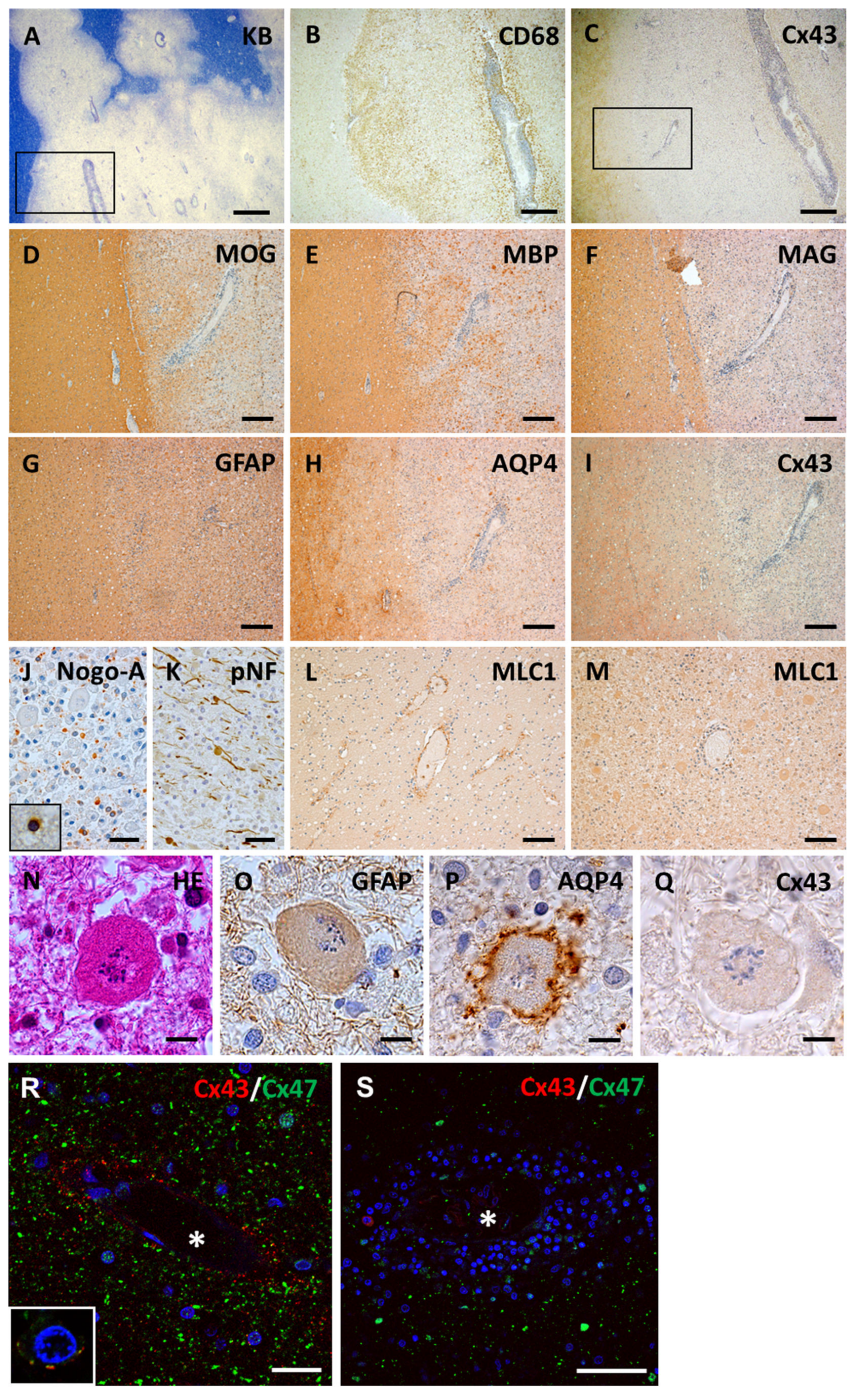
Neuropathological findings in a case with Marburg’s variant of MS (case MS-6). Huge, active demyelinating lesions with perivascular lymphocytic cuffing (A) and infiltrating foamy macrophages phagocytosing myelin debris (B, corresponding to Figure 6A, square). Expression of Cx43 is extensively lost in this lesion (C, corresponding to Figure 6A, square). (D–I) Higher magnification view of the square area in Figure 6C. Loss of MAG is more obvious than for other myelin proteins including MBP and MOG (D–F). Numerous GFAP-positive hypertrophic astrocytes covering active lesions show patchy loss of AQP4 expression and diffuse loss of Cx43 expression (G–I). This lesion is classified as pattern A for Cx43 and pattern B for Cx47/Cx32. Nogo-A-positive oligodendrocytes are decreased and demonstrate apoptotic nuclear condensation (J, insert). Immunoreactivity for phosphorylated neurofilaments reveals degenerated, transected axons with axonal spheroids in the lesion (K). Immunoreactivity for MLC1 is specifically confirmed in perivascular foot process termini in non-affected white matter (L). This staining pattern is no longer observed in the lesion center and only cell bodies of gemistocytic astrocytes are immunopositive for MLC1 (M). Creutzfeldt astrocytes forming multiple micronuclei show a loss of Cx43 despite the preservation of GFAP and AQP4 (N–Q). Double immunofluorescence staining for Cx43 and Cx47 in an active lesion (R, S). In the unaffected white matter, Cx43 and Cx47 expression is well preserved in the perivascular space and white matter (R). Double staining for Cx43 and Cx47 shows partial juxtaposition or colocalization suggestive of GJ plaque formation around oligodendrocytes (inset). In contrast, immunoreactivity for Cx43 is markedly diminished in the perivascular space of inflamed blood vessels while immunoreactivity for Cx47 is preserved (S). Asterisks indicate vascular lumen (R, S). Scale Bar = 1 mm(A); 500 µm (B, C); 200 µm (D–I); 50 µm (J, K); 100 µm (L, M); 10 µm (N–Q); 20 µm (R); 50 µm (S).

MS-6 case, seronegative for anti-AQP4 antibody as assessed by a sensitive cell-based assay [35], showed huge, active demyelinating lesions with perivascular cuffing of lymphocytes and foamy macrophages that had phagocytosed myelin debris (Figure 7A, B). Immunoreactivity for Cx43 was absent throughout the active lesions (Figure 7C). At higher magnification, loss of MAG was more obvious than for other myelin proteins (Figure 7D–F). Numerous GFAP-positive reactive astrocytes in active lesions showed patchy diminution of AQP4 and diffuse loss of Cx43 (Figure 7G–I). Numbers of oligodendrocytic cell bodies were reduced and showed apoptotic nuclear changes (Figure 7J). Degenerated, transected axons with axonal spheroids were also detected (Figure 7K). Immunoreactivity for MLC1 was observed in perivascular foot process of astrocytes in non-affected white matter whereas this staining pattern was not evident in the lesion center, and only cell bodies of gemistocytic astrocytes were immunopositive for MLC1 (Figure 7L, M). Notably, a number of Creutzfeldt astrocytes also appeared in active lesions, which demonstrated Cx43 loss despite the preservation of GFAP and AQP4 (Figure 7N–Q). Double staining for Cx43 and Cx47 revealed bright, dot-like Cx43 and Cx47 signals in astrocytes and around oligodendrocytes in unaffected white matter (Figure 7R), whereas Cx43 immunoreactivity was markedly diminished in reactive astrocytes in the lesion center and periphery (Figure 7S).

#### Distal oligodendrogliopathy in NMO and MS cases

We observed active demyelinating lesions with distal oligodendrogliopathy in three NMO cases (NMO-2, 4 and 10) and two MS cases (MS-3 and 6), characterized by the preferential loss of MAG rather than other myelin proteins, and apoptotic nuclear changes of oligodendrocytes.

#### Relationship between Cx43 astrocytopathy and distal oligodendrogliopathy

Comparisons of pathological features including coexistent astrocytopathy and distal oligodendrogliopathy between NMO and MS cases are summarized in Table 8. Twenty-six active lesions in five NMO cases (NMO-2, 3, 4, 7 and 10) and one NMOSD case showed astrocytic impairment classified as pattern A or B based on Cx43 expression (Table 5). These lesions represented a diffuse loss of Cx43 and AQP4 expression in highly degenerative astrocytes, whereas preservation or reduction of Cx47 and Cx32 expression was observed. Perivascular neutrophil or eosinophil infiltration was present in 38.4% (10/26) of these lesions. Complement activation products (46.1%, 12/26) or macrophages (42.3%, 11/26) were detected within perivascular areas of the lesions. Eight lesions in three NMO cases (NMO-2, 4 and 10) were accompanied by distal oligodendrogliopathy. Fifteen active lesions from three MS cases (MS-3, 4 and 6) also demonstrated astrocytic changes classified as pattern A or B according to Cx43 expression (Table 7). These lesions were characterized as diffuse loss of Cx43 and patchy loss of AQP4 expressions in gemistocytic astrocytes whereas Cx47 and Cx32 expression was reduced in two-thirds of the lesions and preserved in the rest. Creutzfeldt astrocytes existed in 20% (3/15) of these lesions. Neither perivascular neutrophil nor eosinophil infiltration was observed. Complement activation products were found within macrophages in 20% (3/15) of these lesions, but not in perivascular areas (0/13). Twelve lesions in two MS cases (MS-3 and 6) were accompanied by distal oligodendrogliopathy.

**Table 8:** Pathological features of Cx43 astrocytopathy in active lesions of NMO and MS.

**Pathological features**	**NMO and NMOSD (6 cases, n=26)**	**MS (3 cases, n=15)**
Morphology of astrocytes	Highly degenerative (26/26)	Hypertrophic change (15/15), Creutzfeldt astrocytes (3/15)
Immunoreactivity for		
Cx43	Diffuse loss (26/26)	Diffuse loss (15/15)
AQP4	Diffuse loss (26/26)	Patchy loss (11/15), Diffuse loss (4/15)
Cx47 & Cx32	Preserved (8/26), Reduced (18/26)	Preserved (5/15), Reduced (7/15), Diffuse loss (3/15)
Infiltration of neutrophils/eosinophils	10/26	0/15
Complement deposition within		
Perivascular spaces	12/26	0/15
Foamy macrophages	11/26	3/15
Accompanied with distal oligodendrogliopathy		
MAG loss > loss of other myelin proteins	8/26	12/15
Apoptotic nuclear change	8/26	12/15

AQP4 = aquaporin-4, Cx = connexin, MAG = myelin-associated glycoprotein, MS = multiple sclerosis, NMO = neuromyelitis optica

#### Distribution of distal oligodendrogliopathy

Regional distributions of lesions with distal oligodendrogliopathy in relation to Cx43 expression patterns in NMO and MS cases are summarized in Table 9. These lesions were predominantly distributed in the supraspinal regions, namely the cerebrum and brainstem, while only two lesions were observed in the white matter of the spinal cord in MS-6 case.

**Table 9:** Distribution of distal oligodendrogliopathy in relation to Cx43 expression patterns in active demyelinating lesions with NMO and MS.

**Cx43 expression**	**NMO (3 cases, n=8)**				
**Pattern**	**Cerebrum (n=4)**	**Brainstem (n=4)**	**Cerebellum (n=0)**	**Spinal cord (n=0)**	**Optic nerve (n=0)**
Pattern A	3	2	0	0	0
Pattern B	1	2	0	0	0
Pattern C	0	0	0	0	0
Pattern D	0	0	0	0	0
Pattern N	0	0	0	0	0
**Cx43 expression**	**MS (2cases,n=13)**				
**Pattern**	**Cerebrum (n=5)**	**Brainstem (n=6)**	**Cerebellum (n=0)**	**Spinal cord (n=2)**	**Optic nerve (n=0)**
Pattern A	1	5	0	0	0
Pattern B	3	1	0	2	0
Pattern C	1	0	0	0	0
Pattern D	0	0	0	0	0
Pattern N	0	0	0	0	0

See Table 3 for the definition of Cx43 expression patterns. Any pattern with necrosis (pattern X & N) was included as pattern X.

Cx = connexin, MS = multiple sclerosis, NMO = neuromyelitis optica

#### Relationship between Cx43 astrocytopathy and clinical features

Six of nine MS and NMO/NMOSD cases with extensive Cx43 loss classified as pattern A or B died within two years, while none of eight other patients without Cx43 loss had such a rapidly progressive course; death frequency within two years after disease onset was significantly higher in cases with extensive Cx43 loss compared with cases with no Cx43 loss (66.7% vs. 0%, *P* = 0.0090) (Table 10). All six cases that died within two years after disease onset had extensive astrocytic Cx43 loss, regardless of disease phenotype. In contrast, among eleven cases that died more than two years after disease onset, only two had Cx43 loss. Thus, the frequency of Cx43 loss was significantly higher in patients who died within two years after disease onset than those who died more than two years after disease onset (100% vs. 27.3%, *P* = 0.0090). Moreover, in relapsed patients, eight cases with Cx43 loss showed a tendency of higher annualized relapse rates compared with eight cases without Cx43 loss (2.2 ± 1.2 vs. 1.1 ± 0.7, *P* = 0.0653). The Progression Index in patients with extensive Cx43 loss was six-fold higher than in those without Cx43 loss (12.11 versus 1.95) although the difference was not statistically significant because of the small sample size (*P* = 0.1019). Cx43 loss had no significant correlation with age at onset, sex, disease phenotype, disease duration, clinically estimated sites of lesions (number of involved sites and total lesion number), pathologically determined sites of lesions (number of involved sites and total lesion number) and perivascular deposition of complement.

**Table 10:** Comparison of clinical and pathological features according to Cx43 expression status with NMO and MS.

	**Cx43 expression in active demyelinating lesions**		
	**Extensive Cx43 loss (n = 9 cases)**	**Preserved Cx43 (n = 8 cases)**	***p* value**
Clinical features			
Age at onset, yrs	45.77 ± 17.52	36.75 ± 12.84	0.3598
Sex (male : female)	2 : 7	2 : 6	1.0
NMO : MS	6 : 3	5 : 3	1.0
Disease duration, yrs	4.34 ± 5.93	7.11 ± 5.87	0.1019
Progression index	12.11 ± 11.80	1.95 ± 0.88	0.1019
Death within two years (cases, %)	6/9, 66.7%	0/8, 0%	0.0090*
Annualized relapse rate^a^	2.17 ± 1.24	1.08 ± 0.67	0.0653
Clinically estimated sites of lesions^b^			
Number of involved sites	2.55 ± 0.88	3.12 ± 0.83	0.2035
Total lesion number	8.89 ± 8.43	8.28 ± 2.69	0.2823
Pathologically estimated sites of lesions			
Number of involved sites	3.44 ± 1.13	4.12 ± 0.99	0.1928
Total lesion number	9.00 ± 2.00	7.37 ± 2.72	0.1439
Pathological features			
Distal oligodendrogliopathy (cases, %)	5/9, 55.56%	0/8, 0%	0.0294*

* Fisher’s test *p* < 0.05. Cx = connexin, MS = multiple sclerosis, NMO = neuromyelitis optica. ^a^ One NMOSD case died at the first attack and was excluded. ^b^ Case MS-5 was excluded because the number of clinically estimated sites of involvement was unspecified.

#### Relationship between inflammatory components and Cx43 loss in each MS/NMO lesion

**Figure 8:**
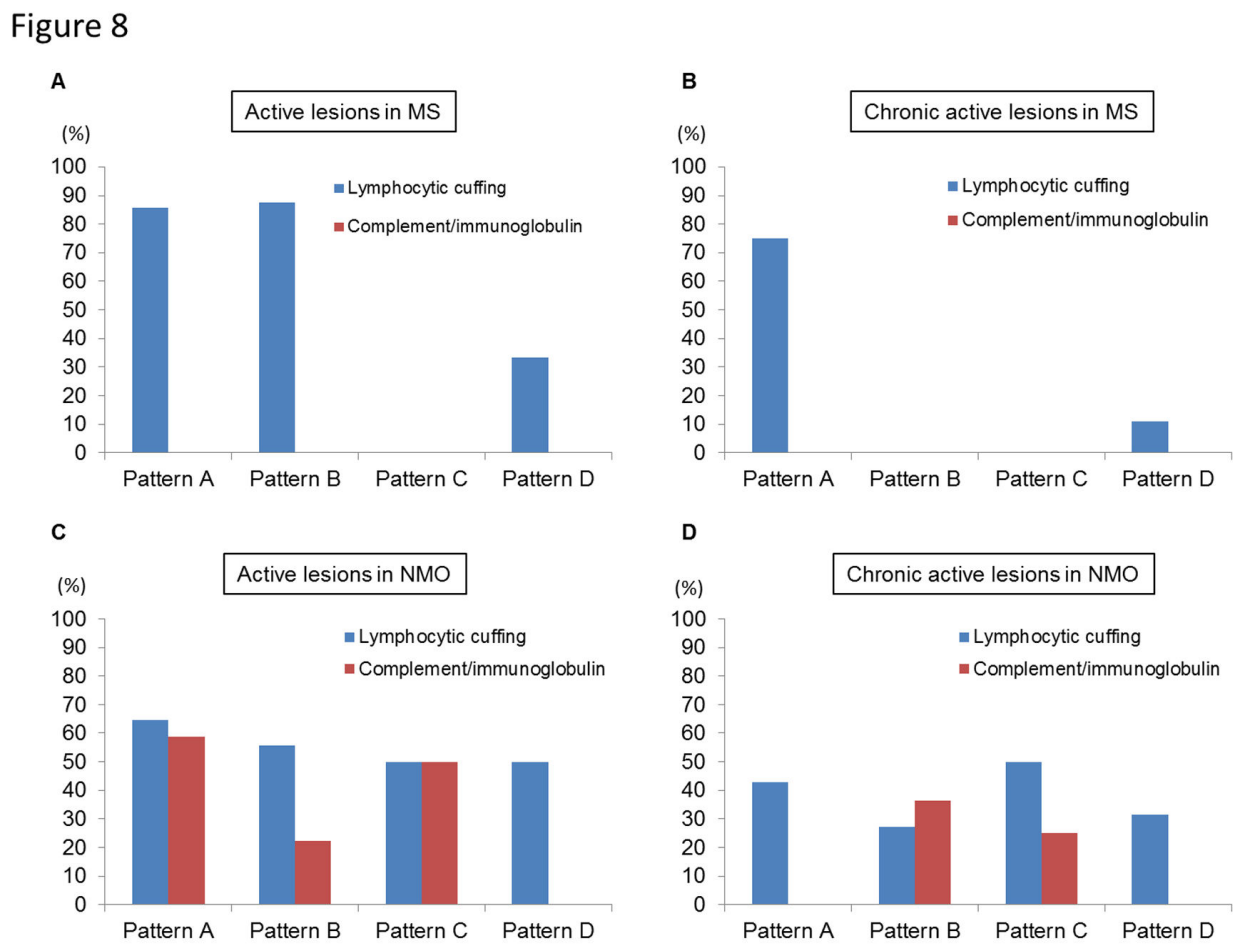
Relationship of inflammatory components with patterns of Cx43 loss in MS and NMO lesions. (A, B) Positivity rates of perivascular lymphocytic cuffing or perivascular deposition of complement and immunoglobulin in active (A) or chronic active (B) lesions of MS cases according to Cx43 patterns. (C, D) Positivity rates of perivascular lymphocytic cuffing or perivascular deposition of complement and immunoglobulin in active (C) or chronic active (D) lesions of NMO/NMOSD cases according to Cx43 patterns. Patterns N lesions are excluded in this figure.

In active and chronic active lesions of MS, perivascular lymphocytic cuffing mainly consisting of T cells was significantly more frequently observed in Pattern A or B lesions (16 of 22 lesions, 72.7%) than in Pattern C or D (2 of 14 lesions, 14.3%) (*P* = 0.0016) while perivascular complement and immunoglobulin deposition was not detected in any lesions (Figure 8A, B). In contrast, in active and chronic active lesions of NMO/NMOSD, perivascular deposition of complement and immunoglobulin was significantly associated with Pattern A or B lesions (16 of 44 lesions, 36.4%) compared with Pattern C or D (2 of 29 lesions, 6.9%) (*P* = 0.0050), while perivascular lymphocytic cuffing was similar in all lesions (Figure 8C, D). However, the mean frequencies of complement and immunoglobulin deposition were only 37.1% of total active lesions and 12.2% of total chronic active lesions.

#### Anti-Cx43 antibody status in patients with NMO and MS

Sera from all patients with NMO, MS, AM, other neurological disorders and healthy controls were negative for anti-Cx43 antibody.

## Discussion

In the present study, systematic immunohistopathological analysis of autopsied MS and NMO cases demonstrated a widespread loss of astrocytic and oligodendrocytic Cxs in CNS tissues. In agreement with previous cohort studies suggesting respiratory diseases are a major cause of death among MS patients [36,37], we believe that the causes of death of MS/NMO patients in this study could be regarded as MS-related deaths. The main new findings of the current study were as follows: 1) half of MS and NMO/NMOSD cases showed preferential loss of astrocytic Cx43 beyond the demyelinated areas in actively demyelinating and chronic active lesions, where heterotypic Cx43/Cx47 astrocyte-oligodendrocyte (A/O) gap junctions were extensively lost, but not in chronic inactive lesions. 2) Cx43 loss (Cx43 astrocytopathy) was significantly associated with a rapidly progressive disease course culminating in death. Annualized relapse rates also tended to be higher in cases with Cx43 astrocytopathy compared with those without. 3) Distal oligodendrogliopathy characterized by selective MAG loss with preservation of other myelin proteins coexisted exclusively in a fraction of Cx43-negative actively demyelinating lesions, but not in Cx43-preserved active lesions. Such lesions were more frequently found in the supraspinal regions, namely the cerebrum and brainstem, but also occasionally in the spinal cord. 4) Oligodendrocytic Cx32 and Cx47 expression was also lost in most lesions from all MS and NMO/NMOSD cases throughout the active to chronic inactive stages, and more extensively in chronic lesions beyond the demyelinated areas. 5) Antibodies against Cx43 were not present in MS or NMO/NMOSD patients. However, this study had some limitations. Autopsied materials were only available for immunohistochemical studies, since it was extremely difficult to obtain freshly frozen CNS tissues from MS and NMO/NMOSD cases in Japan. Nonetheless, the present study contains the largest Asian sample size reported to date, using a variety of targets for immunostaining. Therefore, we believe that the present findings may have significant relevance for a greater understanding of the mechanisms of Asian MS and NMO.

### Loss of astrocytic Cx43 in active NMO and MS lesions

Half of NMO cases showed a pronounced loss of Cx43, almost in parallel with the diminution of AQP4 in active lesions, whereas oligodendrocytic Cx47, a major partner of astrocytic Cx43 in heterotypic A/O GJs [38–41], and other myelin proteins including MAG, Cx32, OSP, MBP and MOG were still preserved. Thus, impairment of astrocytic proteins AQP4 and Cx43 that function in perivascular endfeet may precede demyelination in a considerable number of NMO patients. In NMO, anti-AQP4 antibody-mediated AQP4 loss is suggested to be a primary pathogenic mechanism [7]. However, the mechanism of subsequent demyelination following AQP4 loss remains unclear. As AQP4-deficient mice never develop spontaneous demyelination [20], but rather show an amelioration of EAE [21], the loss of astrocytic endfeet proteins other than AQP4, such as Cx43, may be involved in secondary demyelination in NMO.

In half of the MS cases, we also observed a loss of perivascular astrocytic foot process proteins including Cx43, AQP4 and MLC1, whereas GFAP-positive gemistocytes were abundantly present in active lesions. Gemistocytes in active MS lesions had patchy staining or were negative for AQP4, in accord with previous reports of ours and others [15,16,18]. Intriguingly, loss of Cx43 immunoreactivity occurred more diffusely than that of AQP4 in these lesions. Moreover, in the Marburg’s variant MS case, numerous Creutzfeldt astrocytes showed up-regulation of AQP4 on cell surface membranes, whereas immunoreactivity of Cx43 was totally lost in these cells. Diminution of MLC1, a specific marker for astrocytic foot processes, in active MS lesions may also imply a disruption of astrocytic foot processes in the development of huge lesions, such as Marburg’s lesions. Previously, we demonstrated the cerebrospinal fluid level of angiotensin produced and secreted from astrocyte endfeet was significantly decreased in NMO [42] and MS [43,44] patients, which also suggests that loss of astrocyte endfoot proteins and damage to astrocyte foot processes may occur in acute MS and NMO lesions.

Importantly, in the present study, Cx43 loss was preferentially observed in both MS and NMO cases that had a very short and rapidly progressive disease course resulting in death within two years after disease onset. It is interesting to note that widespread Cx43 loss beyond the demyelinated areas is also seen in Baló’s disease that has an acute aggressive disease course with huge brain lesions [22]. Thus, Cx43 astrocytopathy seems to relate and contribute to acuteness and aggressiveness of the demyelinating disease. Some NMO cases showed an aggressive disease course while others had a benign course, even in the presence of anti-AQP4 antibody [45–47]. The molecular basis for such heterogeneity is currently unknown, but our study suggests that the occurrence of Cx43 astrocytopathy may relate to a more aggressive disease course. Because Cx43 loss was associated with perivascular lymphocyte cuffing in MS and perivascular immunoglobulin and complement deposition in NMO/NMOSD respectively, inflammatory components might have direct effects on the clinical disease course and Cx43 loss could be caused by bystander mechanisms. In either case, the detection of Cx43 loss in biopsy materials, for example in a case of tumefactive MS, may imply an aggressive disease course, suggesting the use of intensive immunotherapy.

### Coexistence of Cx astrocytopathy and distal oligodendrogliopathy

In the present study, we observed that both MS and NMO lesions occasionally showed preferential MAG loss rather than loss of MBP, MOG, OSP, Cx32 and Cx47. Moreover, oligodendrocytes were markedly decreased in these lesions, and the remaining oligodendrocytes underwent apoptotic nuclear changes, which may correspond to “pattern III” lesions or distal oligodendrogliopathy [48,49]. Preferential MAG loss is thought to be an early neuropathological sign of oligodendrocyte dysfunction and has been reported in different conditions, including acute MS, Baló-like lesions, progressive multifocal leukoencephalopathy (PML) and acute hypoxic conditions [48,49]. Interestingly, Brück et al. [50] recently reported that early NMO lesions showed oligodendrocyte apoptosis associated with a selective loss of MAG in addition to typical NMO features, such as the loss of AQP4. In this report, a subset of supraspinal lesions from anti-AQP4 antibody-seropositive NMO patients demonstrated complement activation products within macrophages, oligodendrocyte apoptosis and a preferential loss of MAG. The authors claimed that such pathological features resembled those of MS lesion patterns II and III [50]. Our present observations are in accord with those findings [50]. However, a novel point of our study is that distal oligodendrogliopathic lesions in both NMO and MS exclusively coexisted with Cx43 astrocytopathy, characterized by the diffuse loss of Cx43, suggesting a close correlation between the two pathologies. Cx43/Cx30 double knockout mice showed a pronounced loss of CC1-positive mature oligodendrocytes and increased terminal deoxynucleotidyl transferase dUTP nick end labeling (TUNEL)-positive apoptotic cells [23]. Therefore, disruption of A/O GJ caused by loss of Cx43 may lead to a loss of oligodendrocytes via apoptosis, resulting in secondary demyelination in both MS and NMO. Of note, such coexistence of Cx43 astrocytopathy and distal oligodendrogliopathy was not confined to the supraspinal regions, but occasionally developed in the spinal cord.

### Loss of oligodendrocytic Cx32 and Cx47 in NMO and MS

In MS, Markoullis et al. [26] recently reported that oligodendrocytic Cx32 and Cx47 were extensively diminished, whereas Cx43 showed modest increases in chronic active and inactive lesions and in normal-appearing white matter by quantitative immunoblotting, immunohistochemistry and real-time PCR analyses. We found a profound reduction of oligodendrocytic Cx32 and Cx47 in actively demyelinating lesions in MS and confirmed the loss of oligodendrocytic Cx32 and Cx47 and up-regulation of astrocytic Cx43, presumably resulting from astrogliosis, in chronic active and inactive lesions. For the first time we revealed similar changes also occurred in NMO lesions. Both conditions tended to have a more widespread loss of Cx32 and Cx47 beyond the demyelinated areas in the chronic stage, suggesting that even though demyelinating inflammation had subsided, the loss of oligodendrocyte Cx may not be restored, and may even progress in chronically demyelinated lesions.

### Possible mechanisms of Cx loss

Although MS and NMO commonly have a loss of astrocytic and oligodendrocytic Cxs expression, the triggering factors may be different between MS and NMO. In NMO, anti-AQP4 antibodies could be responsible for the loss, while the mechanism is still unclear in MS. Srivastava et al. [51] recently reported that autoantibodies against Kir 4.1, a potassium channel localized in astrocyte endfeet, is present in nearly half of MS patients and may be a candidate for the loss of astrocytic and oligodendrocytic Cxs expression. Accordingly, autoimmune attacks on astrocyte foot processes may lead to the down-modulation of Cx43, even in MS. However, in the present study, antibodies against astrocytic Cx43 were not observed in MS or NMO patients. In MS, Cx43 loss was significantly associated with perivascular cuffing of lymphocytes mainly composed of T cells. Therefore, instead of autoantibodies, infiltrating T cell secretory factors such as proinflammatory cytokines and chemokines might act on astrocytes to cause Cx43 loss. Because perivascular complement and immunoglobulin deposition was observed in only a fraction of NMO cases, the idea that other inflammatory factors from lymphocytes might contribute to Cx43 loss may not be thoroughly excluded. Recently, a significant reduction of Cx32 and Cx47 was observed in active lesions of MOG-induced EAE [52]. In MOG- and MBP-induced EAE [53], astrocytic Cx43 was also diminished in active lesions, suggesting that myelin antigen-specific T cells could potentially down-modulate Cx expression in oligodendrocytes and astrocytes.

### Possible roles of Cxs loss in demyelinating lesion formation

The disruption of A/O GJ connectivity caused by loss of astrocytic Cx43/Cx30 leads to secondary demyelination and loss of oligodendrocytes in Cx43/Cx30 double knockout mice [23] and lipopolysaccharide (LPS)-induced CNS inflammation [18] models. Thus, the widespread loss of astrocytic Cx43 in the early stages of inflammatory lesion formation partly contributes to subsequent demyelination through disruption of astrocyte-oligodendrocyte interactions. Notably, Ezan et al. [54] demonstrated that mice lacking Cx43/Cx30 in GFAP-positive astrocytes displayed astrocyte endfeet edema and a partial loss of AQP4. Furthermore, the absence of astroglial Cx43/Cx30 weakened the BBB, where astroglial Cx43/Cx30 is indispensable for maintaining BBB integrity [54]. Therefore, loss of astroglial Cx43 may exacerbate demyelinating disease by A/O GJ disruption leading to distal oligodendrogliopathy, and through dysregulation of BBB function, which may explain the rapidly progressive course and higher frequency of relapses in MS and NMO patients with Cx43 loss.

Preserved oligodendrocytic Cx47 in active lesions and preserved or up-regulated astrocytic Cx43 in chronic active and inactive lesions may form hemichannels in the absence of counterparts, i.e., astrocytic Cx43 in active lesions and oligodendrocytic Cx47 in chronic lesions, respectively. Such hemichannels could be hazardous to other cells by secreting toxic substances, such as glutamate [55,56] or to themselves by efflux of potassium ions and influx of calcium and sodium ions [40,57,58]. Therefore, disruption of glial syncytium via A/O GJ channel loss may exert deleterious effects at the acute and chronic stages of inflammatory demyelinating disease.

### Homogeneity and heterogeneity among demyelinating conditions

Our present and previous studies indicate that common pathological features such as Cx43/AQP4 astrocytopathy, Cx32/Cx47 oligodendrogliopathy and distal oligodendrogliopathy, exist between NMO, MS, and Baló’s disease. All acute lesions from Baló’s cases uniformly show such homogeneous pathology. However, MS and NMO cases demonstrate heterogeneity in Cx43/AQP4 astrocytopathy and distal oligodendrogliopathy. The appearance of Cx43/AQP4 astrocytopathy and distal oligodendrogliopathy was partly dependent on lesion stage, emerging in active and chronic active stages, but not during the chronic inactive stage. However, even in acute lesions from the same individual, heterogenous Cx43/AQP4 astrocytopathy and distal oligodendrogliopathy was observed between lesions. In contrast, oligodendroglial Cx32 and Cx47 loss was uniformly seen in NMO, MS, and Baló’s lesions throughout active to chronic inactive stages. Thus, careful evaluation of such pathology is required to determine whether it is disease- or stage-specific, or even common among demyelinating conditions. Considering all of these confounding factors and based on our present and previous findings, we propose that the widespread loss of astrocytic and oligodendrocytic Cxs is prevalent among cases of NMO, MS, and Baló’s disease. Therefore, therapeutic strategies designed to limit damage to A/O GJ channels or restore disrupted A/O GJ channels are warranted.

## Supporting Information

Figure S1 **Expression pattern of Cx32, Cx47 and Cx43 in normal appearing regions of optic nerve tissue in a case of SPG2.**
Cross sections of the optic nerve show expression of Cx32 and Cx47 along myelin sheaths and on the surface of astrocytes adjacent to the myelin sheaths (A, C). Longitudinal sections demonstrate expression of Cx32 and Cx47 around the interfascicular oligodendrocytes (B, D) and at cell-cell interfaces (arrows). Immunoreactivity for Cx43 is observed in astrocytes, perivascular foot processes and around the interfascicular oligodendrocytes (E, F). Scale Bar = 20 µm (A, C, E), 10 µm (B, D, F).
(TIF)

Figure S2 **Double immunostaining for oligodendrocytic Cx32/Cx47 and GFAP in perivascular lesions of pons tissue (case NMO-4).**
Normal perivascular areas of the pons show abundant expression of Cx32 and Cx47 (A, C). Affected perivascular lesions demonstrate relatively preserved expression of Cx32 and Cx47 (B, D) whereas GFAP-positive astrocytic foot processes are markedly degenerated. Scale Bar = 20 µm (A–D).
(TIF)
