# Supplementary figures and images for: Connexin 43 Astrocytopathy Linked to Rapidly Progressive Multiple Sclerosis and Neuromyelitis Optica
Source: PLoS One. 2013 Aug 22;8(8):e72919. doi: 10.1371/journal.pone.0072919 (PMC3749992; doi:10.1371/journal.pone.0072919)

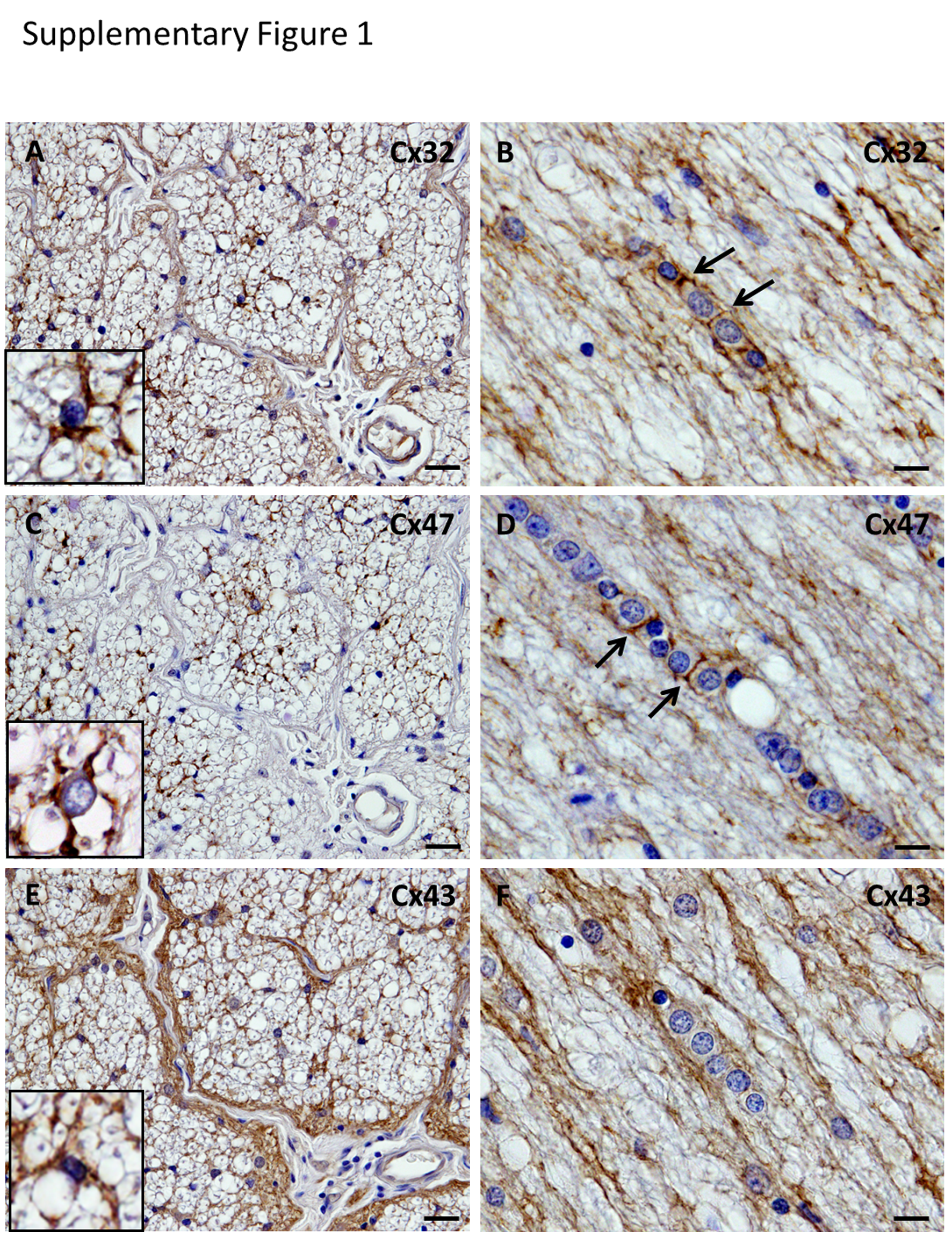

Supplement: Figure S1 — Expression pattern of Cx32, Cx47 and Cx43 in normal appearing regions of optic nerve tissue in a case of SPG2. Cross sections of the optic nerve show expression of Cx32 and Cx47 along myelin sheaths and on the surface of astrocytes adjacent to the myelin sheaths (A, C). Longitudinal sections demonstrate expression of Cx32 and Cx47 around the interfascicular oligodendrocytes (B, D) and at cell-cell interfaces (arrows). Immunoreactivity for Cx43 is observed in astrocytes, perivascular foot processes and around the interfascicular oligodendrocytes (E, F). Scale Bar = 20 µm (A, C, E), 10 µm (B, D, F). (TIF) [file pone.0072919.s001.tif]

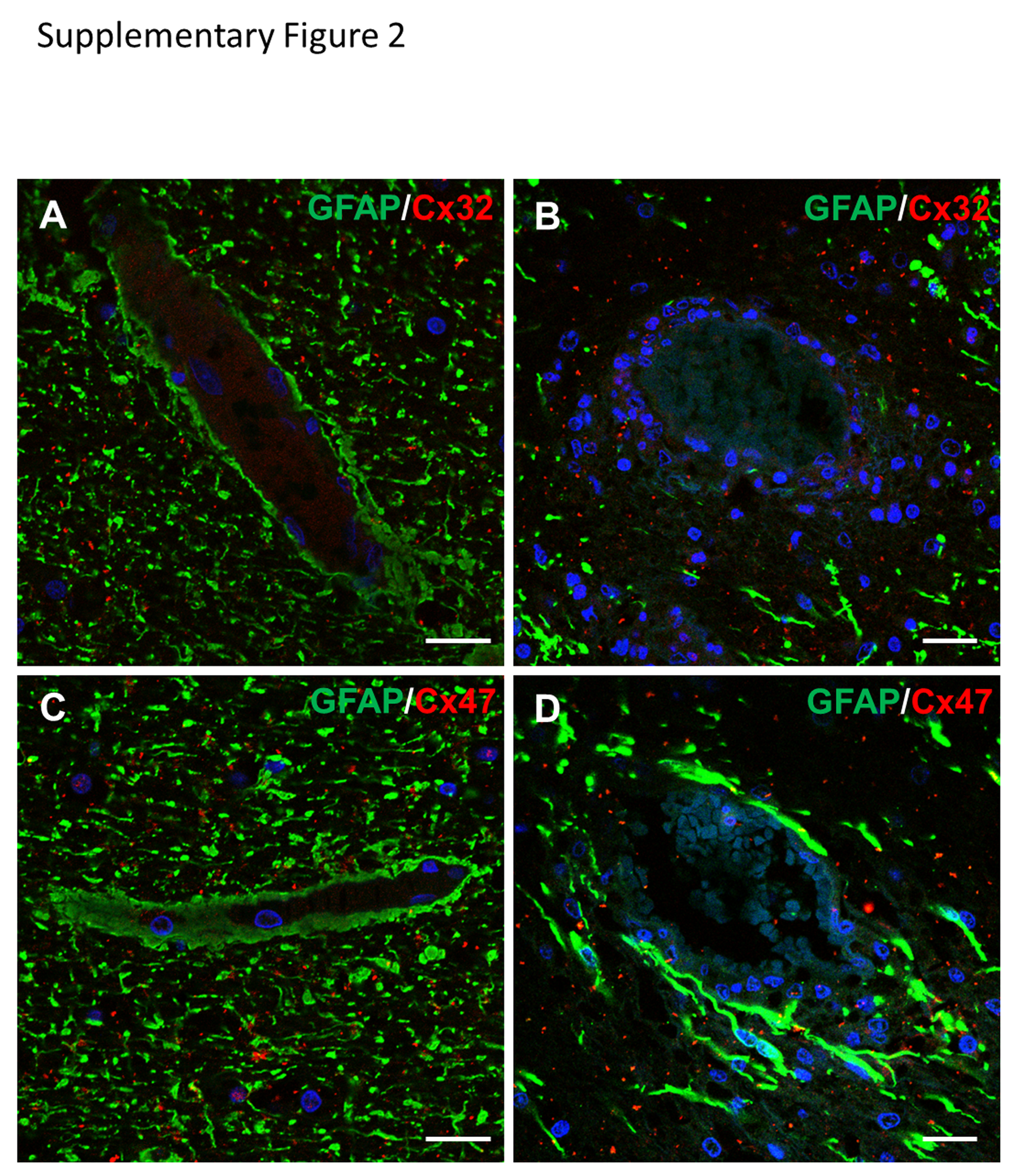

Supplement: Figure S2 — Double immunostaining for oligodendrocytic Cx32/Cx47 and GFAP in perivascular lesions of pons tissue (case NMO-4). Normal perivascular areas of the pons show abundant expression of Cx32 and Cx47 (A, C). Affected perivascular lesions demonstrate relatively preserved expression of Cx32 and Cx47 (B, D) whereas GFAP-positive astrocytic foot processes are markedly degenerated. Scale Bar = 20 µm (A–D). (TIF) [file pone.0072919.s002.tif]
